# Mathematical modeling and biochemical analysis support partially ordered calmodulin-myosin light chain kinase binding

**DOI:** 10.1016/j.isci.2023.106146

**Published:** 2023-02-04

**Authors:** Melissa J.S. MacEwen, Domnita-Valeria Rusnac, Henok Ermias, Timothy M. Locke, Hayden E. Gizinski, Joseph P. Dexter, Yasemin Sancak

**Affiliations:** 1 Department of Pharmacology, University of Washington, Seattle, WA 98195, USA; 2 Data Science Initiative and Department of Human Evolutionary Biology, Harvard University, Cambridge, MA 02138, USA

**Keywords:** Biochemistry, Biochemical mechanism, In silico biology

## Abstract

Activation of myosin light chain kinase (MLCK) by calcium ions (Ca^2+^) and calmodulin (CaM) plays an important role in numerous cellular functions including vascular smooth muscle contraction and cellular motility. Despite extensive biochemical analysis, aspects of the mechanism of activation remain controversial, and competing theoretical models have been proposed for the binding of Ca^2+^ and CaM to MLCK. The models are analytically solvable for an equilibrium steady state and give rise to distinct predictions that hold regardless of the numerical values assigned to parameters. These predictions form the basis of a recently proposed, multi-part experimental strategy for model discrimination. Here we implement this strategy by measuring CaM-MLCK binding using an *in vitro* FRET system. Interpretation of binding data in light of the mathematical models suggests a partially ordered mechanism for binding CaM to MLCK. Complementary data collected using orthogonal approaches that assess CaM-MLCK binding further support this conclusion.

## Highlights

• Calmodulin (CaM) activation of MLCK is key to many cellular functions
• An experimental strategy can distinguish between three models of CaM-MLCK binding
• Experimental data support a partially ordered CaM-MLCK binding mechanism

## Graphical abstract

**Figure:**
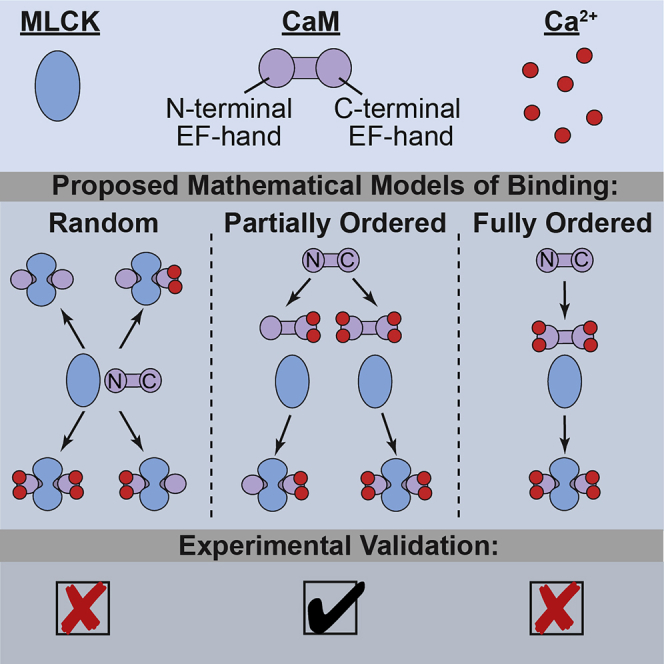

## Introduction

Calmodulin (CaM) is a calcium-binding protein found ubiquitously in the cytosol of eukaryotic cells. CaM contains two globular domains joined by a flexible linker. The N-terminus and C-terminus domains each have a pair of EF-hand motifs and are each capable of binding two calcium ions (Ca^2+^).^1^ CaM acts as a regulator or effector in numerous cellular processes, ranging from muscle contraction to glycogen metabolism and synaptic plasticity.^2^^,^^3^^,^^4^ The Ca^2+^-mediated binding of CaM and either smooth muscle myosin light chain kinase or nonmuscle myosin light chain kinase (henceforth collectively referred to as “MLCK”) is central to functions such as vascular smooth muscle contraction and cell motility. CaM-MLCK binding takes place as follows: Ca^2+^ enters the cytosol and binds to the four Ca^2+^ binding sites of CaM, leading to a dramatic change in CaM protein conformation.^5^ CaM then binds to the CaM-binding domain of MLCK, triggering a conformational change in MLCK that activates the kinase by displacing an autoinhibitory sequence from the kinase’s catalytic domain.^6^^,^^7^ Finally, activated MLCK phosphorylates the 20-kDa regulatory light chains of myosin II, resulting in contraction caused by myosin cross-bridges moving along actin filaments.

**Figure 1:**
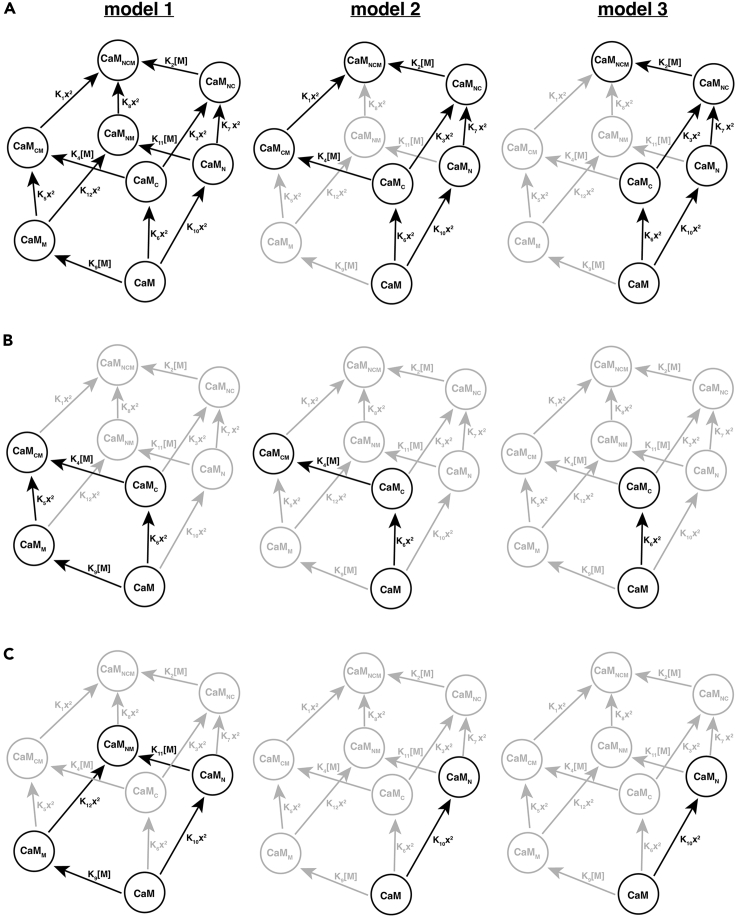
Network diagrams for the binding of Ca^2+^ and MLCK to CaM (A–C) Model 1 is the unordered model proposed by Brown *et al*.^8^ and Fajmut, Brumen *et al*.^9^; models 2 and 3 correspond to the partially and fully ordered models of Fajmut, Jagodic *et al*.^10^ and Kato *et al*.*,*^11^ respectively. (A) shows the networks for CaM^WT^, (B) for CaM^21A,57A^, and (C) for CaM^94A,130A^. Models 2 and 3 are truncations of Model 1; omitted edges and vertices are shown in gray. In each network *x* denotes [Ca^2+^], and *M* denotes MLCK. The subscripts N and C indicate Ca^2+^ binding at the N-terminus and C-terminus EF hands of CaM, respectively, and the subscript M indicates MLCK binding. The figure is adapted from Dexter *et al.*^12^

Multiple formal models have been proposed for the activation of MLCK by Ca^2+^ and CaM.^8^^,^^9^^,^^10^^,^^11^ Given the cooperative nature of Ca^2+^ binding at both the C- and N-terminus of CaM, each of these models treats CaM as having two Ca^2+^-binding sites. The model of Brown *et al*.^8^ and Fajmut, Brumen *et al*.^9^ makes no further assumptions, giving rise to an eight-state reaction network in which MLCK may bind to CaM before or after Ca^2+^ (“Model 1” in Figure 1A). Other previously proposed models are truncations of this network, in which the binding of Ca^2+^ and MLCK is either partially^10^ or fully^11^ ordered (“Model 2” and “Model 3” in Figure 1A, respectively). Accordingly, Model 1 corresponds to a fully random binding mechanism (Ca^2+^ and MLCK can bind to CaM in any order), Model 2 to a partially ordered mechanism (MLCK can bind to CaM after Ca^2+^ is bound at the C-terminus), and Model 3 to a fully ordered mechanism (MLCK can bind to CaM only after Ca^2+^ is bound at both the C-terminus and N-terminus).

In recent work, Dexter *et al*. showed that this class of models is analytically solvable for a CaM/MLCK system at both thermodynamic equilibrium and steady state and that each model predicts distinct steady-state behavior in certain Ca^2+^ concentration regimes.^12^ Importantly, these predictions hold regardless of the numerical values assigned to the model parameters (i.e., the equilibrium constants in Figure 1A), allowing us to develop a strategy for model discrimination that does not require extensive parameter estimation.

**Figure 2:**
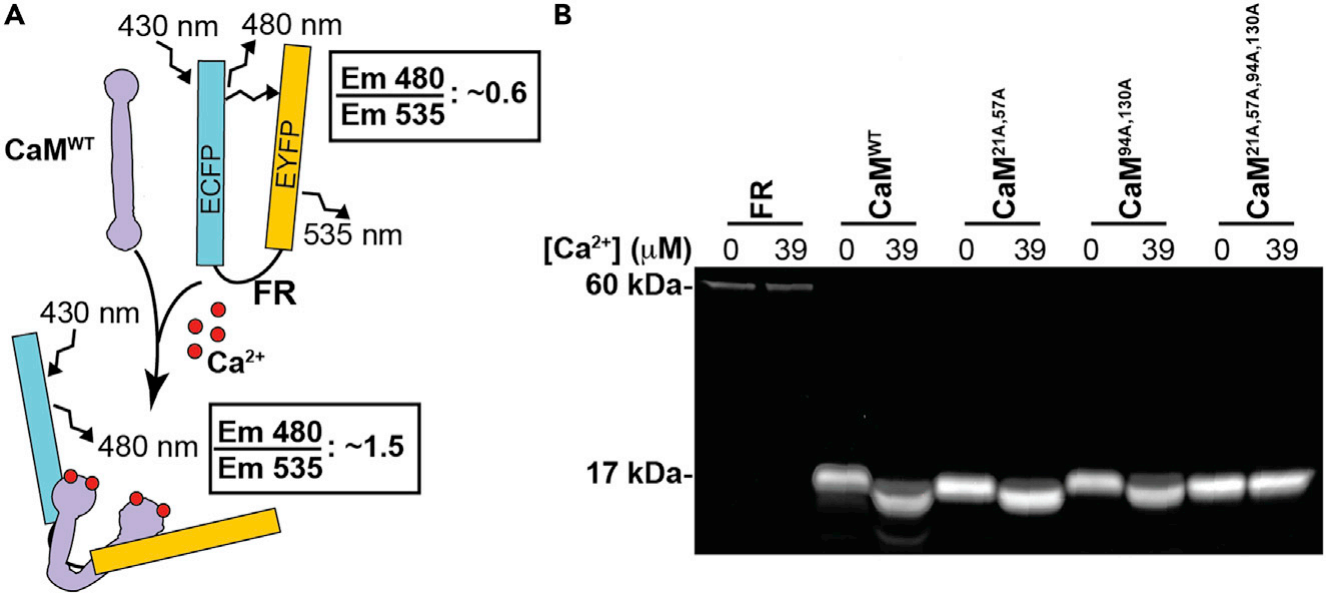
MLCK-based FRET assay principle and purified proteins used in the FRET assay (A) Schematic showing assay principle. In the presence of Ca^2+^, CaM binds to the CaM-binding domain of FR, leading to a decrease in FRET and reduced emission at 535 nm. (B) Representative gel image of purified FR and CaM proteins visualized with SYPRO Ruby protein gel stain following SDS-PAGE. Samples were incubated in buffers containing either 0 μM Ca^2+^ or 39 μM free Ca^2+^ before SDS-PAGE. 8 μg of each CaM protein and 800 ng of FR was loaded.

Our biochemical approach to testing the predictions of the models centers on the use of an established fluorescence resonance energy transfer (FRET) reporter that we term FR. FR has been employed to characterize CaM- and Ca^2+^-dependent activation of MCLK both *in vivo* and *in vitro.*^13^^,^^14^^,^^15^^,^^16^^,^^17^^,^^18^^,^^19^ FR acts as a stand-in for the wild-type MLCK protein; it is composed of an EYFP and ECFP FRET pair, which are linked by the CaM-binding domain of smooth muscle MLCK.^14^ Binding of CaM to FR interferes with FRET, enabling analysis of CaM-FR binding based on the ratio of EYFP and ECFP emission (F480/F535) after excitation at 430 nm (Figure 2A). For our FRET-based binding assays, we measure CaM-FR binding across a wide range of [Ca^2+^] for wild-type CaM (CaM^WT^) and three different CaM mutants: CaM with its N-terminus EF-hands mutated (D21A and D57A mutations, CaM^21A,57A^), CaM with its C-terminus EF-hands mutated (D94A and D130A mutations, CaM^94A,130A^) and CaM with all of its EF-hands mutated (D21A, D57A, D94A, D130A, CaM^21A,57A,94A,130A^). Each of the site-directed Asp to Ala mutations we performed has been demonstrated to prevent Ca^2+^ binding, while having only a slight impact on the structure of each CaM EF hand.^20^

We use a multi-step strategy to discriminate between these models. We first compare Model 1 to Models 2 and 3, and we then compare Model 3 to Models 1 and 2. During the first step, CaM-FR binding at zero [Ca^2+^] is determined as Model 1, but not Models 2 and 3, predicts binding under these conditions. In the second step, the binding of FR to a CaM with impaired N-terminus Ca^2+^ (CaM^21A,57A^) is measured at high [Ca^2+^]. Models 1 and 2, but not Model 3, predict binding between FR and this mutant CaM at high [Ca^2+^]. Finally, we use a CaM mutant with impaired C-terminus Ca^2+^ binding (CaM^94A,130A^) to evaluate additional predictions of Model 2, including the absence of binding of FR to this mutant in high [Ca^2+^]. The predictions about binding are based on algebraic calculations with all parameters treated symbolically; as such, they do not depend on fitting the models to experimental data.

Our binding measurements using the FR falsify key predictions of both Model 1 (random binding) and Model 3 (fully ordered binding) but are consistent with multiple distinct predictions of Model 2, which strongly suggests that CaM-MLCK binding follows a partially ordered mechanism. We further validate our key findings and the utility of the FR system using orthogonal experimental techniques, as well as the measurement of binding between full-length MLCK-FLAG protein and CaM.

## Results

For all binding experiments, we purified either FLAG-tagged FR or MLCK from HEK 293T cells after transient transfection, and purified His-tagged CaM^WT^ and CaM mutants (CaM^21A,57A^ CaM^94A,130A^ and CaM^21A,57A,94A,130A^) after bacterial expression. Gel electrophoresis and SYPRO Ruby staining of purified proteins (Figure 2B), as well as mass spectrometry analysis (Figure S1), established that the protein preparations were free of major contaminants. It is well documented that the electrophoretic mobility of CaM in SDS-PAGE changes following Ca^2+^ binding-induced conformational change; this gel mobility shift is commonly used as a readout of Ca^2+^-CaM binding.^21^^,^^22^^,^^23^^,^^24^ We observed an expected Ca^2+^-dependent gel mobility shift in our CaM^WT^, CaM^21A,57A^ and CaM^94A,130A^ protein preparations (Figure 2B),^25^ but observed no gel mobility shift for FR or CaM^21A,57A,94A,130A^ in the presence of Ca^2+^. These data suggest that the purified CaM proteins each retain their expected Ca^2+^ binding affinity.

**Figure 3:**
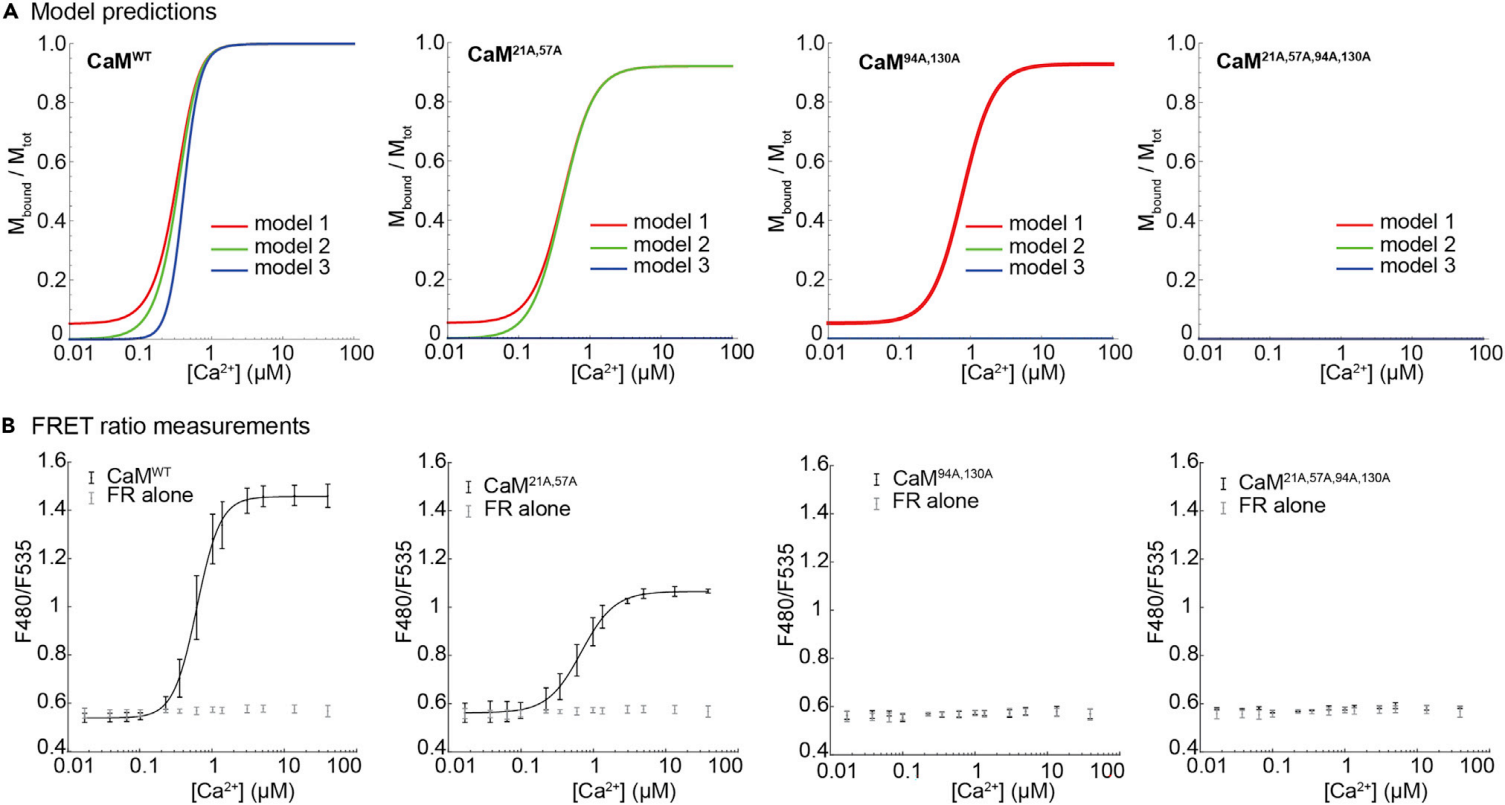
Predictions of mathematical models of MLCK binding and FRET measurements in the presence of excess CaM relative to FR (A) Theoretical CaM-MLCK binding curves for CaM^WT^, CaM^21A,57A^ CaM^94A,130A^ and CaM^21A,57A,94A,130A^ The predicted curves were calculated assuming [CaM] = 0.713 μM and [MLCK] = 0.0237 μM (the concentrations of CaM and FR used in the main experiments) and the reference parameter values from Fajmut, Brumen *et al.*^9^ (B) Experimental binding curves showing FRET ratio measurements of CaM proteins. For the experiments, FR was incubated either alone, or with a 30-fold excess of CaM^WT^, CaM^21A,57A^ CaM^94A,130A^ or CaM^21A,57A,94A,130A^, in the presence of indicated [Ca^2+^] in 96-well plates. Emission at 480 nm and 535 nm was measured after excitation at 430 nm using a plate reader. The [Ex 430, Em 480] fluorescence intensity was then divided by the [Ex 430, Em 535] intensity to yield an emission ratio, which is plotted. Shown are mean ± SD of 13 replicates for FR only, 15 replicates for CaM^WT^, 16 replicates for CaM^21A,57A^, 8 replicates for CaM^94A,130A^, and 3 replicates for CaM^21A,57A,94A,130A^

Having validated our experimental tools, we collected FRET-based CaM-FR binding data to use in our model discrimination strategy. The previous theoretical analysis identified a two-part strategy for distinguishing between the three models of CaM-MLCK binding, which is described in detail in Dexter *et al.*^12^ The analysis involves deriving algebraic expressions for the fraction of total MLCK (*F*) that is predicted to bind to CaM as a function of free [Ca^2+^] for each of the models. Plots of *F* are shown in Figure 3A for CaM^WT^, CaM^21A,57A^ CaM^94A,130A^ and CaM^21A,57A,94A,130A^ (assuming the reference values for the equilibrium constants compiled by Fajmut, Brumen *et al*.^9^ and the concentrations of CaM and MLCK used in our experiments). The model discrimination strategy rests on two predictions that differ between the three models and that hold true regardless of the specific numerical values assigned to the model parameters, such as the equilibrium constants in Figure 1. The first is that only Model 1 predicts non-zero binding of MLCK in zero [Ca^2+^], with the fraction bound given by the following expression:

(Equation 1) $$F_{1}^{WT}\left(0\right)=\frac{2K_{9}{CaM}_{tot}}{1+K_{9}\left({CaM}_{tot}{+M}_{tot}\right)+\sqrt{1+2K_{9}\left({CaM}_{tot}+M_{tot}\right)+K_{9}^{2}{\left({CaM}_{tot}-M_{tot}\right)}^{2}}}\text{,}$$

where CaM_tot_ and MLCK_tot_ denote total CaM and total MLCK, respectively. The second is that only Model 3 predicts zero binding of MLCK to a CaM mutant with nonfunctional N-terminal EF hands, such as CaM^21A,57A^ at any free [Ca^2+^]. In contrast, both Models 1 and 2 predict non-zero binding, with the fraction in the high-[Ca^2+^] limit given by

(Equation 2) $$\lim_{\left[Ca^{2+}\right]\to \infty }F_{1}^{N}=\lim_{\left[Ca^{2+}\right]\to \infty }F_{2}^{N}=\frac{2K_{4}CaM_{tot}}{1+K_{4}\left(CaM_{tot}+M_{tot}\right)+\sqrt{1+2K_{4}\left(CaM_{tot}+M_{tot}\right)+K_{4}^{2}{\left(CaM_{tot}-M_{tot}\right)}^{2}}}.$$

The algebraic structure of Equations 1 and 2 is identical; the only difference between the two expressions is which equilibrium constant appears (K_9_ in Equation 1, K_4_ in Equation 2). As discussed in Dexter *et al*.*,*^12^ this similarity is a consequence of the fact that the reaction network reduces to a bimolecular reaction in both the low- and high-[Ca^2+^] limits.

To test these predictions experimentally, we used a plate reader to detect changes in CaM-FR binding as a function of free [Ca^2+^] with a 30-fold molar excess of CaM^WT^, CaM^21A,57A^, CaM^94A,130A^, or CaM^21A,57A,94A,130A^ relative to FR. [CaM] was 673 nM (13 ng/μL), and [FR] was 22.9 nM (1.3 ng/μL). After collecting Ex 430/Em 480 and Ex 430/Em 535 data from different CaM-FR pairs (Figure S2), we divided each [Ex 430/Em 480] fluorescence intensity value by its corresponding [Ex 430/Em 535] value to yield a ratio, which is plotted in Figure 3B. We confirmed the presence of excess CaM compared to FR, and equal protein amount across experiments, by SDS-PAGE and SYPRO Ruby staining of the samples after FRET measurement (Figure S3).

For the first model discrimination test, we compared the FRET ratio of the CaM^WT^-FR pair with baseline in zero and low [Ca^2+^]. Contrary to the prediction of Model 1, we find no evidence that the signal in zero [Ca^2+^], or the area under the binding curve for 0 μM ≤ [Ca^2+^] ≤ 0.038 μM, is higher than baseline (p = 0.994 and p = 0.995, respectively, by a one-tailed Mann-Whitney *U* test). For the second test, we compared the FRET ratio of CaM^21A,57A^ -FR pair in high [Ca^2+^]. We found that the FRET ratio in the maximum concentration used (39 μM [Ca^2+^]) is significantly higher than baseline (p = 2.83∗10^−6^ by a one-tailed Mann-Whitney *U* test), as is the area under the complete binding curve (p = 2.83∗10^−6^ by a one-tailed Mann-Whitney *U* test). These observations are sufficient to falsify Model 3, which predicts zero binding of MLCK to any CaM mutant with impaired N-terminus Ca^2+^ binding. As such, only the predictions of Model 2 are consistent with the full set of experimental results. As an additional test of Model 2, we examined the binding of C-terminus mutant CaM^94A,130A^ in high [Ca^2+^]; consistent with the predictions of the model, we found no significant difference over baseline for the FRET ratio in 39 μM [Ca^2+^] or for the area under the complete binding curve (p = 0.345 and p = 0.294, respectively, by a one-tailed Mann-Whitney *U* test).

A striking feature of the experimental binding curves is the significant difference in maximum binding between CaM^WT^ and CaM^21A,57A^ (p = 1.16∗10^−6^ by a one-tailed Mann-Whitney *U* test). This difference is also consistent with the predictions of Model 2 (Figure 3A). Assuming Model 2, the fraction of CaM^21A,57A^ bound in the high-[Ca^2+^] limit is given by Equation 2. For CaM^WT^, the limiting expression is the same as Equation 2 but with K_2_ instead of K_4_ (for the reasons explained above), so that the model predicts equal maximum binding if K_2_ = K_4_. In the reference parameter set, K_2_ = 1,000 and K_4_ = 16.7, corresponding to predicted binding of 99.9% for CaM^WT^ and 92.0% for CaM^21A,57A^ in 39 μM [Ca^2+^]. As such, the experimentally observed difference is also predicted by our mathematical analysis, assuming that the literature parameter values are correct within an order of magnitude.

The validity of our experimental approach for model discrimination depends on the ability of the CaM-FR interaction to accurately mirror CaM-MLCK binding with sufficient sensitivity, as well as other considerations related to the experimental perturbations. To address these potential limitations, we performed a series of control experiments and tested the robustness of our experimental design and data.

First, to confirm that the FRET ratio of FR is only altered by the binding of Ca^2+^-bound CaM, we collected 460 nm-700 mm emission spectra with Ex 430 of the FR in 0 and 39 μM [Ca^2+^]. When FR was assayed alone, FR showed no changes in FRET ratio as a function of [Ca^2+^] and produced fluorescence peaks at Em 480 and Em 535 (Figure S4A). Incubating FR with either CaM^WT^ or N-terminus mutant CaM^21A,57A^ in the presence of 39 μM [Ca^2+^] caused FR fluorescence to increase at ∼ Em 480 and decrease at ∼ Em 535 (Figures S4B and S4C). No change to FR fluorescence was observed in the absence of Ca^2+^ at any wavelength following FR incubation with CaM^WT^ or N-terminus mutant CaM^21A,57A^. C-terminus mutant CaM^94A,130A^ did not noticeably change the FR spectra at any wavelength, with or without Ca^2+^ (Figure S4D).

**Figure 4:**
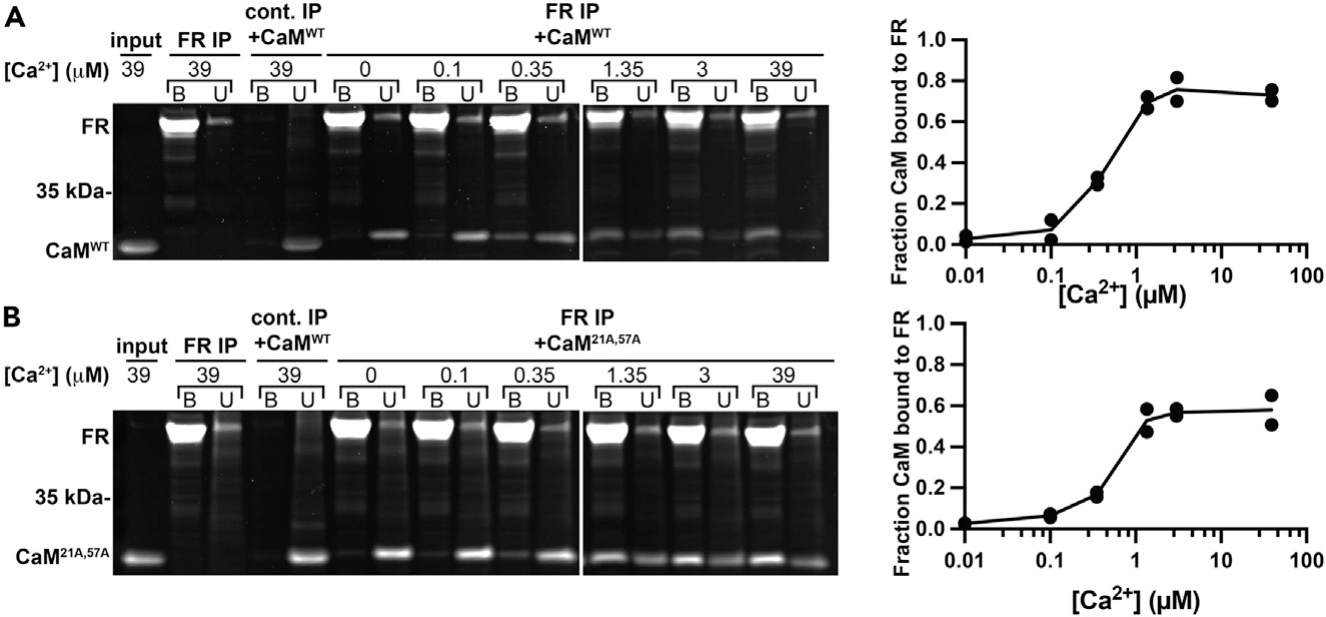
Purified CaM^WT^ and CaM^21A,57A^ show Ca^2+^-dependent interaction with FR (A and B) Representative images showing binding between bead-immobilized FR and (A) CaM^WT^ or (B) CaM^21A,57A^ in buffers with the indicated free [Ca^2+^]. Unbound and bound fractions were analyzed by SDS-PAGE followed by SYPRO Ruby protein gel stain. Fraction of CaM bound to the FR was quantified using ImageJ. The binding graph shows the quantification of two experimental replicates; curve shows the average of the two data points at each [Ca^2+^]. FR without CaM addition and a binding experiment using non-transfected HEK 293T cells are controls. The input lane shows the purified CaM^WT^ or CaM^21A,57A^ added to the on-bead binding assay.

Having seen no interactions between FR and CaM^94A,130A^ or CaM^21A,57A,94A,130A^ during our FRET-based binding assays, we wanted to confirm that the interactions we observed between CaM^WT^-FR and CaM^21A,57A^-FR are specific, Ca^2+^-dependent, and representative of binding between CaM and MLCK. To do so, we performed several on-bead binding assays using either FR or MLCK-FLAG. When bead-bound FR was incubated in 50 μL of buffer with 505 nM CaM^WT^ (approximately 20 ng/μL), the fraction of CaM^WT^ bound to FR increased proportionally with free [Ca^2+^]. At 39 μM [Ca^2+^], the majority of the CaM^WT^ input was bound to the FR (Figure 4A). When the same experiment was repeated using N-terminus mutant CaM^21A,57A^, more than half of CaM^21A,57A^ bound to FR at 39 μM [Ca^2+^] (Figure 4B). These results suggest that the purified CaM proteins are functional and bind to Ca^2+^ and the FR, and that there is negligible non-specific binding between the FR and the purified CaM proteins. To determine if CaM-FR binding is representative of binding between CaM and full-length MLCK, we performed an on-bead binding assay after transient expression and purification of MLCK-FLAG protein. Despite the presence of several background bands, we observed the same CaM binding pattern for both MLCK-FLAG and FR. At 39 μM [Ca^2+^], the majority of the CaM^WT^ input appeared bound to MLCK-FLAG (Figure S5A), and there was a partial binding of N-terminus mutant CaM^21A,57A^ to MLCK-FLAG (Figure S5B). There was no apparent interaction between C-terminus mutant CaM^94A,130A^ and MLCK-FLAG at 39 μM [Ca^2+^] (Figure S5C). None of the CaM proteins bound to MLCK-FLAG at 0 μM [Ca^2+^].

We also investigated the relationship between the “fraction bound” calculated in the modeling analysis and the FRET ratio we use as a proxy for CaM-FR interaction. Although the Em 480/Em 535 ratio may not have a one-to-one relationship to “fraction bound,” the Em 480/Em 535 ratio correlates closely with CaM-FR binding at high and low [Ca^2+^], as calculated using on-bead binding assays (Figure S6). Moreover, the same FRET ratio has been shown to mirror MLCK phosphorylation *in vivo.*^14^ As a result, we conclude that the ratio can be used to characterize CaM-FR interaction for our model discrimination strategy, which rests on differential binding predictions in zero and high [Ca^2+^].

**Figure 5:**
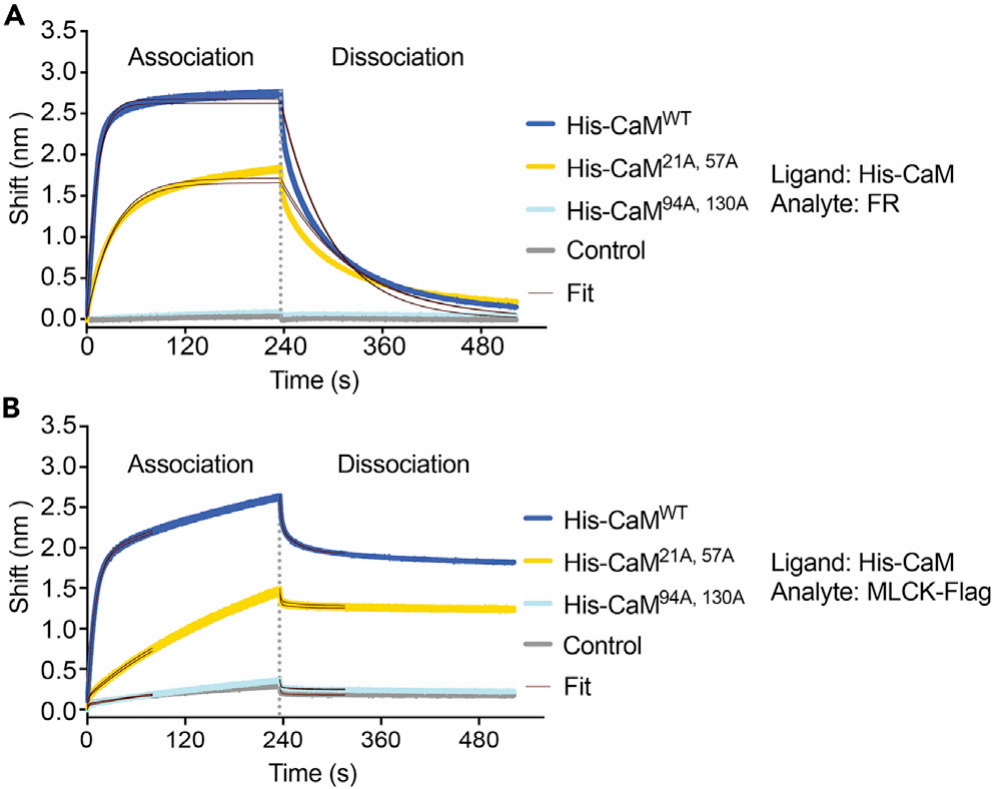
Bio-Layer Interferometry (BLI) binding assays show no binding between FR or MLCK-FLAG and CaM^94A,130A^ (A and B) BLI was performed using purified His-tagged calmodulin (CaM^WT^, CaM^21A,57A^, or CaM^94A,130A^) as a ligand immobilized to a Ni-NTA biosensor and either (A) FR or (B) MLCK-FLAG as an analyte. Two replicates of each condition are shown. “Control” refers to the analyte binding to the ligand-free probe. Data were analyzed using the Octet data analysis software (Octet Analysis Studio).

We do not observe binding between FR and C-terminus mutant CaM^94A,130A^ in our FRET-based binding assays. To investigate the possibility that CaM^94A,130A^ might bind to the FR but fail to interfere with FRET, we used a Bio-Layer Interferometry (BLI) assay as an orthogonal method to measure binding between FR and CaM. Binding detection in the BLI assay is independent of FRET measurement, which enabled us to decouple binding and FRET interference. The BLI assay showed robust binding between FR and CaM^WT^ at high [Ca^2+^], an intermediate degree of binding between FR and CaM^21A,57A^ at high [Ca^2+^], and no detectable binding between FR and CaM^94A,130A^ at high [Ca^2+^] (Figure 5A). Performing a BLI assay using CaM^WT^, CaM^21A,57A^, and CaM^94A,130A^ with purified MLCK-FLAG yielded comparable results (Figure 5B).

We next assessed the intrinsic sensitivity of the FRET assay. To test if our assay can detect small changes in binding, which is necessary for many of the comparisons involved in our model discrimination analysis, we measured FRET at 39 μM [Ca^2+^] with different FR:CaM ratios. Assuming Model 2 and the reference parameter values from Fajmut, Brumen *et al*.*,*^9^ if the FR:CaM molar ratio is increased from 2:1 to 2:1.2, the amount of CaM-bound FR in 39 μM [Ca^2+^] is predicted to increase by 8.5% (from 46.2% bound to 54.7% bound). When we repeated our FRET-based CaM-FR binding assay using these ratios (22.9 nM FR and 11.45 nM or 13.75 nM CaM), we observed a significant increase in F480/F535 (Figure S7) (p = 0.00029 by a one-tailed Mann-Whitney *U* test), with a calculated 8.7% difference in FRET ratios between the two conditions. We therefore conclude that, under our experimental conditions, the assay is sensitive enough to detect at least a 9% increase in the fraction of CaM-bound FR.

The predictions of zero MLCK binding in a particular [Ca^2+^] hold for any set of numerical values assigned to the model parameters (the equilibrium constants K_1_ … K_11_ in Figure 1), as shown in Dexter *et al.*^12^ For predictions of non-zero binding, however, the magnitude of binding does depend on the choice of parameters. As such, it is theoretically possible that the model could predict a binding fraction that is non-zero but too small to detect experimentally in an important concentration regime. To address this potential concern, we undertook a sensitivity analysis of the two key predictions of non-zero MLCK binding. For numerical calculations with the models, we used the set of reference parameter values compiled by Fajmut, Brumen *et al*. from previous biochemical studies.^9^ As shown in Equation 1, the prediction of Model 1 for the fraction of MLCK bound in zero [Ca^2+^] depends on the value of a single parameter (K_9_). Assuming the parameter value selected by Fajmut, Brumen *et al*.^9^ based on several prior studies (K_9_ = 0.078 μM^−1^) and the concentrations of CaM^WT^ and FR used in the main experiment, the model predicts 5.2% binding in zero [Ca^2+^]. As is clear from the structure of Equation 1, the fraction of MLCK predicted to bind in zero [Ca^2+^] increases with the ratio of total CaM to total MLCK. To confirm our falsification of Model 1, we therefore repeated the binding experiment in zero [Ca^2+^] with a much higher [CaM] (35.65 μM), for which 73.5% binding is predicted with the reference parameter values and 21.7% binding is predicted with K_9_ = 0.0078 μM^−1^ (i.e., 10-fold lower than the reference value). As in the main experiment, the FRET ratio did not increase above baseline in zero [Ca^2+^] (Figure S8), providing strong evidence against Model 1 even if previous parameter estimates are incorrect by an order of magnitude (p = 0.998 by a one-tailed Mann-Whitney *U* test).

For Model 2, the fraction of MLCK predicted to bind to the N-terminus mutant CaM^21A,57A^ depends on two parameters, K_4_ and K_6_ (Equation 2). We confirmed the robustness of the key prediction of non-zero binding in 39 μM [Ca^2+^] by calculating $F_{2}^{N}$ for 200,000 combinations in which values for each of the two parameters were chosen at random from the interval [0.01v, 100v], where v is the reference value from Fajmut, Brumen *et al.*^9^ Across the combinations $F_{2}^{N}$ was never less than 18%, a level of binding straightforward to distinguish from baseline; the full distribution is shown in Figure S9.

Finally, we confirmed that the addition of the purified proteins to the prepared Ca^2+^ buffers did not significantly alter the buffers’ free [Ca^2+^]. We measured the fluorescence intensity of two Ca^2+^ indicator dyes with different Ca^2+^ affinities (Fluo-4 and Calcium Green), before and after the addition of the buffer in which our CaM proteins were stored. The largest volume of protein added to any of our FRET-based binding experiments was 1.3 μL per assay, so we tested this volume. We did not observe a significant change in indicator dye fluorescence intensity after CaM storage buffer addition, which suggests that any changes to free [Ca^2+^] that are experimentally introduced are smaller than can be detected reliably with Fluo-4, a dye that has a very high Ca^2+^ affinity (17 nM) (Figure S10) (p = 0.23-0.99 by a two-tailed Mann-Whitney *U* test).

These additional experiments and sensitivity analyses show that our experimental system, which includes the well-validated FRET-based binding assay, is robust and rigorously controlled, as outlined here. We find that the FR and CaM proteins we used are free of major contaminants; SYPRO stained SDS-PAGE gels of the CaM proteins also confirm that these proteins display the predicted altered electrophoretic mobility upon binding to Ca^2+^, which strongly suggests that they bind Ca^2+^ as expected. Follow-up plate reader experiments demonstrated that FRET interference is sensitive enough to allow discrimination between candidate CaM-MLCK binding models. We also show that a change in the FRET ratio is caused only by the binding of Ca^2+^-bound CaM proteins; conversely, orthogonal BLI experiments confirm that a lack of FRET interference corresponds to a lack of FR-CaM binding. We further confirmed that the [Ca^2+^] of our experimental buffers is not altered by the addition of our experimental proteins. Finally, on-bead binding experiments with both FR and MLCK-FLAG reproduced the key findings of our FRET-based binding assays, a critical check of the robustness of our experimental system. These data, in combination with the MLCK-FLAG data from our BLI assays, suggest that FR-CaM binding accurately mirrors binding between CaM and full-length MLCK protein, and that our FR data can confidently be used for model discrimination.

In sum, our binding measurements falsify key predictions of both the fully random binding mechanism (Model 1) and the fully ordered binding mechanism (Model 3). Our data instead support Model 2, which assumes partially ordered binding between CaM and MLCK (Figure 1). As predicted by Model 2, we observe zero binding in low [Ca^2+^] for CaM^WT^ and all CaM mutants, non-zero binding in high [Ca^2+^] for CaM^WT^, non-zero binding in high [Ca^2+^] for N-terminus mutant CaM^21A,57A^, and zero binding in high [Ca^2+^] for both of our CaM mutants containing mutations in the C-terminus domain (CaM^94A,130A^ and CaM^21A,57A,94A,130A^). Model 2 makes six correct predictions and no incorrect predictions (Table 1). Model 1 makes two falsified predictions: non-zero binding in low [Ca^2+^] for CaM^WT^, and non-zero binding in high [Ca^2+^] for the C-terminus mutant, CaM^94A,130A^ Finally, Model 3 makes one falsified prediction: zero binding in high [Ca^2+^] for the N-terminus mutant, CaM^21A,57A^.

**Table 1:** Summary of model discrimination results for CaM and MLCK binding

	model 1	model 2	model 3	observed
*zero[Ca*^*2+*^*]*				
CaM^WT^	**B**	NB	NB	NB
CaM^21A,57A^	**B**	NB	NB	NB
CaM^94A,103A^	**B**	NB	NB	NB
*high[Ca*^*2+*^*]*				
CaM^WT^	B	B	B	B
CaM^21A,57A^	B	B	**NB**	B
CaM^94A,103A^	**B**	NB	NB	NB

B denotes binding, NB denotes no binding. Bolded entries indicate experimentally falsified predictions.

## Discussion

In recent work, Dexter *et al*. analyzed a class of previously developed theoretical models of CaM-MLCK binding and proposed a multi-part strategy for distinguishing between them.^12^ Our primary contribution here is an experimental implementation of this model discrimination strategy, which suggests a partially ordered mechanism for binding. Our analysis sheds new light on a controversy that has persisted for several decades and demonstrates a productive interplay between mathematical modeling and biochemical analysis.

It is important to remember that model discrimination strategies of this kind work by process of elimination. Models can be ruled out when their predictions contradict experimental data, but the failure of some models does not guarantee the correctness of others. We present here evidence sufficient to falsify all but one of the models of CaM-MLCK binding in the literature (Table 1). Data collected using wild-type and mutant CaM proteins in three different binding experiments (FRET, BLI, and on-bead binding) in zero and high [Ca^2+^], which used both FR and MLCK-FLAG when possible, rule out both Model 1 and Model 3. Our data are consistent with Model 2, which makes six correct predictions. Taken together, these results strongly support a partially ordered mechanism in which MLCK can bind to CaM after Ca^2+^ is bound at the C-terminus (Model 2).

To investigate MCLK-CaM binding, we centered our experimental design on an FR system that uses the CaM-binding domain of smooth muscle MLCK. Prior studies have demonstrated that this reporter accurately reflects CaM-MLCK binding in a variety of *in vivo* and *in vitro* contexts.^13^^,^^14^^,^^15^^,^^16^^,^^17^^,^^18^^,^^19^ Moreover, our FRET-based CaM-FR binding assay enabled the collection of robust and reproducible data using purified CaM and FR proteins, with minimal risk of interference from endogenous proteins or contaminants. This system is also amenable to complementary, non-FRET-based biochemical techniques to assess CaM-FR binding, which allowed us to confirm the FRET-based binding data we obtained. Although the FR we used has been extensively validated as a good proxy for CaM-MLCK binding, the use of an MLCK fragment still does raise concerns about the behavior of the fragment relative to the full-length protein. To allay these concerns, we repeated two key experiments—on-bead binding experiments and BLI—with a full-length MLCK-FLAG protein in lieu of the FR protein. In each of these experiments, FR and MLCK-FLAG performed similarly, showing the utility of the FRET-based binding assay for our model discrimination strategy.

In HEK 293T lysates, changing myosin phosphorylation has been shown to correspond to changes in the FRET ratio produced by FR. Changes to the FRET ratio can therefore be used as a proxy for MLCK activation.^14^ It is important to note that changes in the FRET ratio do not necessarily correspond 1:1 with changes to the fraction of FR bound to CaM across the full range of [Ca^2+^] tested. Determining the proportionality constant, however, is not necessary for our model discrimination strategy, which relies primarily on FRET ratios measured at 0 μM [Ca^2+^] and 39 μM [Ca^2+^]. We also complement this FRET-based binding assay data with on-bead binding assays showing that in 39 μM [Ca^2+^], the majority of CaM^WT^ binds to either FR or full-length MLCK protein (MLCK-FLAG) expressed using human smooth muscle MLCK gene *MYLK1*, while a negligible amount of CaM binds to FR or MLCK-FLAG in 0 μM [Ca^2+^]. We therefore conclude that FRET ratio data represent CaM^WT^-MLCK binding in 0 μM [Ca^2+^] and 39 μM [Ca^2+^].

It should be noted that mammalian myosin light chain kinases are a group of serine/threonine kinases encoded by at least four genes: *MYLK1*, *MYLK2*, *MYLK3*, and *MYLK4*.^26^ *MYLK1* has alternative initiation sites that enable the expression of at least four protein products including nonmuscle (long isoform) MLCK and smooth muscle (short isoform) MLCK. *MYLK2* encodes an MLCK isoform expressed solely in skeletal muscle, *MYLK3* encodes a cardiac-specific MLCK (MLCK3), and the gene product(s) of *MYLK4* remain largely uncharacterized.^25^^,^^26^ All MLCK proteins, except MLCK4, have a CaM-binding peptide that shows sequence homology to both the peptide used in our FR, which is derived from avian smooth muscle MLCK, and to the CaM-binding domain of our MLCK-FLAG protein. As a result, we expect that our findings here can be generalized to other MLCK proteins and their interaction with CaM and Ca^2+^.

In addition to providing a blueprint for future model discrimination efforts that integrate mathematical and biochemical approaches, our work may also prove useful in a translational or pharmaceutical context, as MLCK and its activation by CaM have been linked to the pathogenesis of human disease. For example, increased organization of the sarcomere, the contractile unit of the striated muscle, is observed during the onset of cardiomyocyte hypertrophy. CaM-activated MLCK has been shown to mediate sarcomere organization induced by a hypertrophic agonist in cultured cardiomyocytes and *in vivo.*^27^ Fuller characterization of the Ca^2+^-CaM-MLCK interaction network may therefore prove relevant for future drug discovery efforts.

### Limitations of the study

Although our data consistently support partially ordered binding between MLCK and CaM, there are meaningful limitations to our study. Our experimental strategy does not investigate MLCK activation in response to CaM-Ca^2+^ binding; binding data should not be interpreted as evidence of MCLK activation. In addition, MLCK is only one of the many binding partners of CaM; future work is needed to determine the binding mechanisms of other proteins regulated by CaM-Ca^2+^.

## STAR★Methods

### Key resources table

**Table:** 

REAGENT or RESOURCE	SOURCE	IDENTIFIER
**Bacterial and virus strains**		

BL21(DE3)*Escherichia coli* bacteria	New England BioLabs	cat. no. C2527I

**Chemicals, peptides, and recombinant proteins**		

DMEM	Thermo Fisher Scientific	cat. no. 11-965-118
Glutamax	Fisher Scientific	cat. no. 35-050-061
PBS	Thermo Fisher Scientific	cat. no. 20012050
Penicillin/streptomycin solution	VWR	cat. no. 45000-652
Fetal bovine serum	Life Technologies	cat. no. 26140087
SDS	Sigma-Aldrich	cat. no. L4509-1KG
BME/2-mercaptoethanol	Sigma-Aldrich	cat. no. M3148-25ML
Glycerol	Sigma-Aldrich	cat. no. G5516-1L
Tris–HCl	Sigma-Aldrich	cat. no. T5941
Bromophenol Blue	VWR	cat. no. 97061-690
10X Tris/Glycine Buffer	Boston BioProducts	cat. no. BP-150-4L
Tris-Glycine 12% Gel, 10-well or 15-well	Bio-Rad	cat. no. 4561043 or 4561046
Methanol	Sigma-Aldrich	cat. no. 32213-2.5L
Acetic Acid	Sigma-Aldrich	cat. no. A6283-500ML
Transfection reagent; X-treme (GENE) 9	Sigma-Aldrich	cat. no. 6365779001
SYPRO™ Ruby Protein Gel Stain	Thermo Fisher Scientific	cat. no. S12000
HEPES	Sigma-Aldrich	cat. no. H3375-1KG
KOH	Sigma Millipore	cat. no. 1050121000
NaCl	Sigma-Aldrich	cat. no. 746398-5KG
Ethylenediaminetetraacetic acid (EDTA)	Sigma-Aldrich	cat. no. 607-429-00-8
Triton X-100	Sigma-Aldrich	cat. no. X100-1L
Protease Inhibitor Tablets; Complete™, Mini, EDTA-free Protease Inhibitor Cocktail	Sigma-Aldrich	cat. no. 5892953001
Anti-FLAG affinity gel	Sigma-Aldrich	cat. no. A2220-5ML
3X FLAG peptide	Sigma-Aldrich	cat. no. F4799-4MG
Low-salt LB broth	Millipore Sigma	cat. no. L3397-1KG
Isopropyl beta-D-1-thiogalactopyranoside (IPTG)	Sigma-Aldrich	cat. no. I5502-5G
Imidazole	Sigma-Aldrich	cat. no. I5513
EGTA	Sigma-Aldrich	cat. no. 324626-25GM
TCEP	Sigma-Aldrich	cat. no. C4706-2G
Calcium calibration buffer kit	Invitrogen	cat. no. C3008MP
Calcium Green 5N, Hexapotassium Salt, Cell Impermeant	Thermo Fisher Scientific	cat. no. C3737
Fluo-4 Cell Impermeant	Thermo Fisher Scientific	cat. no. F14200
Chloroacetamide	Sigma	cat. no. C0267
TCEP	Sigma-Aldrich	cat. no. C4706-2G
Rappsilbers Stage tipping Paper (23), C-18 material; CDS Empore C18 Extraction Disks	Fisher	cat. no. 13-110-016
Formic acid for HPLC LiChropur	Sigma	cat. no. 5438040100
Trifluoroacetic acid (TFA), HPLC Grade, 99.5+%	Alfa Aesar	cat. no. AA446305Y
Acetonitrile (ACN)	Sigma-Aldrich	cat. no. 271004-100ML
Triethylammonium bicarbonate buffer 1.0 M, pH 8.5±0.1	Sigma	cat. no. T7408
Ethanol for HPLC	Sigma	cat. no. 459828
Trypsin	Promega	cat no. V5111
Methanol for HPLC	Sigma	cat. no. 494291
Bovine Serum Albumin (BSA)	Sigma-Aldrich	cat. no. A3294-100G
Calcium chloride	Sigma-Aldrich	cat. no. 746495-1KG

**Critical commercial assays**		

Genlantis MycoScope PCR Detection Kit	VWR	cat. no. 10497-508

**Experimental models: Cell lines**		

HEK 293T cell line	Acquired from the Sabatini Lab at the Whitehead Institute for Biomedical Research.	This cell line has been published extensively (for example, PMID: 29074583).

**Recombinant DNA**		

His-CaMWT DNA in bacterial expression plasmid	This paper	Plasmid available upon request.
His-CaM21A,57A DNA in bacterial expression plasmid	This paper	Plasmid available upon request.
His-CaM94A,130A DNA in bacterial expression plasmid	This paper	Plasmid available upon request.
His-CaM21A,57A,94A,130A DNA in bacterial expression plasmid	This paper	Plasmid available upon request.
MLCK FRET Reporter (FR) DNA in mammalian expression plasmid	This paper	Plasmid available upon request. FR was first developed by Dr. Anthony Persechini and was cloned in mammalian expression vector; see PMID: 14741352 for reference
Full-length MLCK DNA in mammalian expression plasmid	This paper	Plasmid available upon request. MLCK gene was amplified from Addgene plasmid # 46316, from the Bresnick Lab, and cloned into a mammalian expression vector.

**Software and algorithms**		

Andromeda version 1.5.2.8	MaxQuant	https://www.maxquant.org/
MATLAB R2020a	MathWorks	https://www.mathworks.com/products/new_products/release2020a.html
doseResponse; MATLAB R2020a	MathWorks	https://www.mathworks.com/matlabcentral/fileexchange/33604-doseresponse
Octet® Analysis Studio	Sartorius	https://www.sartorius.com/en/products/protein-analysis/octet-bli-detection/octet-systems-software
Prism 7	Graphpad	https://www.graphpad.com/support/prism-7-updates/
Mathematica 12.1	Wolfram	https://support.wolfram.com/51966
ImageJ	National Institutes of Health	https://imagej.net/ij/index.html

**Deposited data**		
Code for mathematical modeling and statistical analysis	This paper	https://osf.io/zt4rk/

### Resource availability

#### Lead contact

Information and requests for resources and reagents should be directed to and will be fulfilled by the lead contact, Dr. Yasemin Sancak (sancak@uw.edu).

#### Materials availability

All plasmids generated in this study at University of Washington, Seattle, WA, USA are available upon request.

### Experimental model and subject details

HEK 293T cells were acquired from the Sabatini Lab at the Whitehead Institute for Biomedical Research. The identity of the cell line was confirmed using STR analysis; cell line is of female origin. Cells were grown in DMEM supplemented with 1X GlutaMAX and 10% fetal bovine serum. Cells were tested for mycoplasma every three months and were confirmed to be free of mycoplasma contamination. Cells were cultured at 37°C with 5% CO2.

BL21(DE3) *E. coli* bacterial strain was acquired from the Zheng Lab at the University of Washington, Department of Pharmacology solely for the purpose of protein expression. Bacteria are stored as a flash frozen stock at −80C.

### Method details

#### Cell culture

• DMEM; Thermo Fisher Scientific, cat. no. 11-965-118
• Glutamax; Fisher Scientific, cat. no. 35-050-061
• PBS; Thermo Fisher Scientific, cat. no. 20012050
• Penicillin/streptomycin solution; VWR cat. no. 45000-652
• Fetal bovine serum; Life Technologies, cat. no. 26140087
• Genlantis MycoScope PCR Detection Kit; VWR cat. no. 10497-508

#### Gel sample preparation, running, and imaging

##### Reducing sample buffer, pH 6.8

• SDS; Sigma-Aldrich Cat. no. L4509-1 KG
• BME/2-mercaptoethanol; Sigma-Aldrich Cat. no. M3148-25 ML
• Glycerol; Sigma-Aldrich Cat. no. G5516-1L
• Tris–HCl: Sigma-Aldrich cat. no. T5941
• Bromophenol Blue; VWR Cat. no. 97061-690

##### Gel electrophoresis

• 10X Tris/Glycine Buffer; Boston BioProducts Cat. no. BP-150-4L
• Tris-Glycine 12% Gel, 10-well or 15-well; Bio-Rad Cat. No. 4561043 or 4561046

##### Gel imaging

• Methanol; Sigma-Aldrich cat. no. 32213-2.5L
• Acetic Acid; Sigma-Aldrich cat. no. A6283-500 ML

Gels were stained with SYPRO Ruby Protein Gel Stain (#S12000) following manufacturer’s instructions and imaged with iBrightCL 1000 on fluorescent protein gel setting.

#### Expression of FR and MLCK-FLAG via transient transfection

• Transfection reagent; X-treme(GENE) 9, Sigma-Aldrich, cat. no. 6365779001

Day 1: Either 5 million (purification) or 2 million (on-bead binding assays) HEK 293T cells were plated on 15 cm or 10 cm plates, respectively. Day 2: 5 μg FR plasmid (purification), 1.5 μg FR plasmid (on-bead binding assays with FR), or 3 μg MLCK-FLAG plasmid (on-bead binding assays with MLCK-FLAG) was transfected using transfection reagent. *Day 4*: Cells were harvested, and FR or MLCK-FLAG was either purified (see below, “Purification of FR”) or used for on-bead binding assays.

#### Purification of FR and MLCK-FLAG

##### Lysis buffer

• 50 mM HEPES-KOH (HEPES, Sigma-Aldrich, #H3375-1 KG KOH, Sigma Millipore, # 1050121000)
• 150 mM NaCl, Sigma-Aldrich, # 746398-5 KG
• 5 mM EDTA: Sigma-Aldrich, # 607-429-00-8
• 1% Triton X-100: Sigma, #X100-1L
• FLAG Peptide Elution Buffer: 50 mM HEPES, 500 mM NaCl, pH 7.4
• Protease Inhibitor Tablets; Complete, Mini, EDTA-free Protease Inhibitor Cocktail, Sigma-Aldrich cat. no. 5892953001
• Anti-FLAG affinity gel; Sigma-Aldrich, cat. no. A2220-5 ML
• 3X FLAG peptide; Sigma-Aldrich cat. no. F4799-4 MG
• Chromatography spin column; Bio-Rad cat. no. 7326204

Cells were lysed using lysis buffer supplemented with proteases inhibitors. Lysates were triturated in tubes and then centrifuged at 17,000 g for 10 min. Cell supernatant was divided into three tubes with 200 μL of anti-FLAG affinity gel slurry (50:50 bead/lysis buffer). These tubes were rocked at 4 °C for 1 h, and beads were washed three times with lysis buffer. A 22.5-gauge syringe was used to aspirate all remaining liquid from the tubes. FLAG-tagged protein was eluted with 90 μL of elution buffer, and 10 μL of 3XFLAG peptide prepared at 5 mg/mL for 30 min at 30 °C. The gel/elution buffer slurry from all three tubes was then pipetted into one spin column and spun at max speed for 5 min.

#### Expression and purification of wild-type and mutant CaM via bacterial induction

• Low-salt LB broth: Millipore Sigma, #L3397-1 KG
• Isopropyl beta-D-1-thiogalactopyranoside (IPTG): Sigma-Aldrich, #I5502-5G
• HiTrap Q HP anion exchange chromatography column: Cytiva, # 17115301
• Centrifugal protein concentrator, 10K cutoff: Amicon, # UFC8010

##### Lysis buffer

• 20 mM Tris-HCl, Sigma-Aldrich cat. no. T5941
• 100 mM NaCl, Sigma-Aldrich, cat. no. 746398-5 KG
• 20 mM imidazole, Sigma-Aldrich, cat. no. I5513
• 0.4 mM EGTA, Sigma-Aldrich cat. no. 324626-25 GM
• 0.5 mM TCEP, Sigma-Aldrich cat. no. C4706-2G

##### Elution buffer

• 20 mM Tris-HCl, Sigma-Aldrich cat. no. T5941
• 100 mM NaCl, Sigma-Aldrich, cat. no. 746398-5 KG
• 200 mM imidazole, Sigma-Aldrich, cat. no. I5513
• 0.4 mM EGTA, Sigma-Aldrich cat. no. 324626-25 GM
• 0.5 mM TCEP, Sigma-Aldrich cat. no. C4706-2G

##### Buffer A

• 100 mM NaCl, Sigma-Aldrich, cat. no. 746398-5 KG
• 20 mM Tris-HCl, Sigma-Aldrich cat. no. T5941

##### Buffer B

• 1M NaCl, Sigma-Aldrich, cat. no. 746398-5 KG
• 20 mM Tris-HCl, Sigma-Aldrich cat. no. T5941

BL21 (DE3) bacteria transformed with a CaM expression vector were grown at 37 °C, 200 RPM until OD600 reached 0.8–1.0. Cultures were cooled to 18°C and supplemented with IPTG to a final concentration of 0.3 mM before incubation at 18°C, 200 RPM for 18 h. Bacteria were pelleted, frozen, and stored at −80°C until protein purification. Cells were lysed using sonication for 5 min at 40% power before being centrifuged at 17,400 RPM for 40 min to separate the soluble and insoluble portions of the lysate. The clarified lysate was loaded onto a Ni-NTA gravity column and then washed with 10 column volumes of lysis buffer before elution with 5 column volumes of elution buffer. The eluted protein was loaded manually into a 1 mL anion exchange chromatography column. HPLC was performed using buffers A and B. The HPLC fractions that contained the highest concentrations of protein were collected and concentrated to their final concentrations using a centrifugal filter with a 10 kDa molecular weight cutoff. Stock protein concentrations were as follows: CaM^WT^: 3.98 μg/μL; CaM^21A,57A^: 4.96 μg/μL; CaM^94A,130A^: 1.36 μg/μL; CaM^21A,57A,94A,130A^: 1.52 μg/μL. Purified protein was aliquoted, flash frozen in a liquid nitrogen dewar, and then stored at −80 °C until use.

#### FRET-based CaM-FR binding assay

• Calcium calibration buffer kit; Invitrogen cat. no. C3008MP
• Microplate reader; BioTek Synergy H1
• Black 96-well plates; Greiner Bio-One cat. no. 655076

For each CaM-FR binding assay condition, 2 μg of CaM (either wild-type or mutant) and 200 ng FR was thoroughly mixed with 150 μL of Ca^2+^ buffer (14 conditions between 0 μM and 39 μM). 150 μL of the resulting mixture was pipetted into 1 well of a 96-well plate (Greiner Bio-One, # 655076). CaM was used at a final concentration of 673 nM (13 ng/μL); FR was used at a final concentration of 22.9 nM (1.3 ng/μL). All protein preps (FR and CaM) were at sufficiently high concentrations that each protein addition added negligible volume; stock protein concentrations were as follows: CaM^WT^: 3.98 μg/μL; CaM^21A,57A^: 4.96 μg/μL; CaM^94A,130A^: 1.36 μg/μL; CaM^21A,57A,94A,130A^: 1.52 μg/μL; FR: 0.52 μg/μL. Follow-up experiments with Ca^2+^ indicator dyes confirmed that the final [Ca^2+^] were unaffected by the addition of CaM or FR to the Ca^2+^ buffer (see “CaM and Ca2+ buffer with Ca2+ indicator dye assay” below). For functional assay, all 14 Ca^2+^ conditions were tested together, batch-wise. All wells were read at 430, 480 excitation/emission, and then again at 430, 535 excitation/emission using a microplate reader. The [430, 480] read was divided by the [430, 535] read to yield an emission ratio, which was plotted relative to [Ca^2+^]. A small portion of each prepared Ca^2+^ condition was conserved and prepared with sample buffer, to be loaded onto a gel and stained.

#### CaM-FR FRET-Based binding sensitivity assays

• Microcon concentrator column, Millipore cat. No. 42407

To test the robustness of the falsification of Model 1 to uncertainty in parameter values, we repeated the binding assay in zero [Ca^2+^] using 35.65 μM [CaM^WT^]. CaM^WT^ was concentrated to a stock concentration of 66.6 μg/μL using a concentrator column so that a comparable volume of protein stock (relative to previous assays) could be used. The “FRET-based CaM-FR binding assay” was then performed as described above, except using 35.65 μM [CaM^WT^].

To determine a lower sensitivity limit for the CaM-FR binding assay, 22.9 nM FR was added to 150 μL of high [Ca^2+^] buffer along with either 11.45 or 13.74 nM [CaM^WT^], which resulted in a CaM^WT^:FR molar ratio of either 1:2 or 1.2:2. 150 μL of the resulting mixture was then read in a plate reader using the program employed by the “FRET-based CaM-FR binding assay” (see above) and analyzed accordingly.

#### CaM-FR spectral scanning

• Calcium calibration buffer kit; Invitrogen cat. no. C3008MP
• Microplate reader; BioTek Synergy H1
• Black 96-well plates; Greiner Bio-One cat. no. 655076

CaM and FR were added to either 0 or 39 μM [Ca^2+^] as per “FRET-based CaM-FR binding assay” above. CaM was used at a final concentration of 673 nM (13 ng/μL); FR was used at a final concentration of 22.9 nM (1.3 ng/μL). Using an excitation wavelength of 430 nm, the wells were read using spectral scanning between wavelengths 460 nm and 700 nm, with an emission step of 10 nm. The fluorescence intensity of each wavelength was then plotted.

#### CaM-FR and CaM-MLCK-FLAG on-bead binding assays

For assays utilizing FR, assay data were collected under 8 different conditions: FR bound to anti-FLAG M2 affinity gel beads and CaM under 6 different [Ca^2+^] (0 μM, 0.1 μM, 0.35 μM, 1.35 μM, 3 μM, and 39 μM) and two controls (bead-bound FR alone in 39 μM [Ca^2+^]; beads with CaM in 39 μM [Ca^2+^]). For assays utilizing MLCK-FLAG, assay data were collected under 4 different conditions: MLCK-FLAG bound to anti-FLAG M2 affinity gel beads and CaM under 2 different [Ca^2+^] (0 μM and 39 μM) and two controls (bead-bound MLCK-FLAG alone in 39 μM [Ca^2+^]; beads with CaM in 39 μM [Ca^2+^]).

##### Binding assay protocol

Transiently transfected cells were washed once with chilled PBS, and then harvested in 1% Triton buffer using a cell scraper. Cells were triturated to ensure complete lysis, and then were spun down at maximum speed (17,000g) for 10 min using a 4°C centrifuge. 160 μL bead:glycerol M2 FLAG slurry was prepared for FLAG-tagged protein binding by washing three times with 1 mL 1% Triton buffer before being divided evenly between 8 tubes. The cleared lysate from the transiently transfected cells was divided evenly between 7 of the tubes, while cleared lysate from control WT HEK 293T was added to the eighth tube (control lysate). The beads were incubated with the lysate on a rocker at 4°C for 45 min. After incubation, the two control tubes were washed twice with 200 μL 39 μM [Ca^2+^], while the tubes for the 6 different Ca^2+^ conditions were each washed with the appropriate Ca^2+^ buffer. 50 μL of the appropriate Ca^2+^ buffer was then added to each experimental tube, while 50 μL of 39 μM Ca^2+^ was added to each control tube. Finally, for FR assays, 0.5 μg of CaM was added to all tubes except the reporter-only condition; final CaM concentration was approximately 505 nM (10 ng/μL). For MLCK-FLAG assays, 0.25 μg of CaM was added to all tubes except the MLCK-FLAG-only condition; final CaM concentration was approximately 24 nM (5 ng/μL) (NOTE: CaM concentration for MLCK-FLAG assays was decreased to compensate for decreased MLCK-FLAG expression relative to FR expression). CaM stock was at a sufficiently high concentration to ensure the addition of CaM did not meaningfully alter Ca^2+^ buffer concentration (see “CaM and Ca2+ buffer with Ca2+ indicator dye assay below”) (Figure S5). All tubes were incubated at room temperature for 15 min and were flicked occasionally. Following incubation, the tubes were spun for 1 min at 1,000 g to pellet the beads. 40 μL of the unbound portion was conserved and prepared with 10 μL 5X reducing sample buffer. The beads for all conditions were then washed with 50 μL of the appropriate Ca^2+^ buffer and all liquid was aspirated from the beads using a syringe to remove residual unbound CaM. The beads were then incubated with 40 μL of FLAG peptide prepared in elution buffer for 30 min at 30 °C to elute FLAG tagged FR. After elution, the beads were loaded to a spin column and spun for 1 min at 1000 g at room temperature to recover the eluate without the beads. This eluted sample was then boiled with 10 μL 5X reducing sample buffer. Finally, an “input” sample was prepared by adding 0.5 μg of CaM to 40 μL of 39 μM [Ca^2+^] solution and 10 μL 5X reducing sample buffer.

##### Binding quantification

Bands were quantified using ImageJ. A rectangle was drawn around the unbound CaM band in the FR/CaM assay at 0 μM [Ca^2+^]. This same rectangle was used to quantify the mean gray value within the rectangle for each binding condition. A measurement was also taken of the gel background, from the “input” lane containing only calmodulin. The background intensity measurement was subtracted from each quantified region. % binding was calculated using the following equation: % binding = bound fraction/[unbound CaM fraction + bound CaM fraction]. For Figure S6, binding fraction was normalized by setting binding at 0 μM [Ca^2+^] to 0% and at 39 μM [Ca^2+^] to 100% and calculating the % bound in the rest of the samples by normalizing to this scale.

#### CaM and Ca^2+^ buffer with Ca^2+^ indicator dye assay

• Calcium Green 5N, Hexapotassium Salt, Cell Impermeant; Thermo Fisher Scientific, cat. no. C3737
• Fluo-4 Cell Impermeant; Thermo Fisher Scientific, cat. no. F14200
• Microplate reader; BioTek Synergy H1
• Calcium calibration buffer kit; Invitrogen cat. no. C3008MP
• Black 96-well plates; Greiner Bio-One cat. no. 655076

##### CaM storage buffer (“dummy buffer”)

• 250 mM NaCl, Sigma-Aldrich, cat. no. 746398-5 KG
• 20 mM Tris, pH 8, Sigma-Aldrich cat. no. T5941

This assay was performed to confirm that the addition of CaM protein to the Ca^2+^ buffer used for the CaM-FR binding assay did not change the final [Ca^2+^]. Two “sets” of each of the 14 different Ca^2+^ buffers used for CaM-FR binding assay were prepared, with dye (Calcium Green or Fluo-4) added to a final concentration of 1 μM. For one set of buffers, the buffer in which the CaM proteins was stored was added (“dummy buffer”). The amount of “dummy buffer” added was equal in volume to the maximum volume of CaM added per reaction (i.e., the C-terminus mutant CaM was the most dilute CaM prep, and approximately 1.5 μL CaM was added to each assay well, so an equivalent amount of “dummy buffer” was used for these experiments). 150 μL of each of these prepared buffers was added to wells of a black 96-well plate and data were collected using the GFP filter of a plate reader.

##### Mass spectrometry analysis of CaM proteins

• Calcium calibration buffer kit, Invitrogen cat. no. C3008MP
• Chloroacetamide, Sigma cat. no. C0267
• TCEP, Sigma-Aldrich cat. no. C4706-2G
• Rappsilbers Stage tipping Paper (23), C-18 material: CDS Empore C18 Extraction Disks, Fisher cat. no. 13-110-016
• Formic acid for HPLC LiChropur, Sigma cat. no. 5438040100
• Trifluoroacetic acid (TFA), HPLC Grade, 99.5+%, Alfa Aesar, cat. no. AA446305Y
• Acetonitrile (ACN), Sigma-Aldrich cat. no. 271004-100 ML
• Mass spectrometer: LC-MS: Orbitrap Fusion Lumos Tribrid (Thermo Fisher Scientific) and EASY-nLC 1200 System (Thermo Fisher Scientific)
• Triethylammonium bicarbonate buffer 1.0 M, pH 8.5 ± 0.1, Sigma cat. no. T7408
• Ethanol for HPLC, Sigma cat. no. 459828
• Promega trypsin, cat no. V5111
• Methanol for HPLC, Sigma cat. No. 494291

##### StageTip buffer A

• 5% ACN
• 0.1% TFA
• MQ water

##### StageTip buffer B

• 0.1% TFA
• 80% ACN
• MQ water

##### Extraction solution

• 0.1% TFA, diluted in MQ water

##### Running the gel

30 μg of wild-type CaM and N-terminus mutant CaM were each mixed with high Ca^2+^ calibration buffer to a final volume of 20 μL. A blank control consisting of CaM storage buffer and Ca^2+^ calibration buffer was also prepared. All samples were reduced using 1 mM final concentration of TCEP and alkylated using 2 mM final concentration of chloroacetamide (CAM) at 37°C for 20 min on a shaker. They were then quenched using 1 mM final concentration of TCEP at 37°C for 20 min on a shaker. Next, they were mixed with 5X gel loading buffer, boiled for 5 min, and loaded into a 10% gel (see “gel electrophoresis” above). The samples were run until the dye front reached the bottom of the gel. The gel was then rinsed and stained using Coomassie stain for 1 h, and destained overnight.

##### In-gel digestion protocol

The next day, two bands from each lane were excised and cut into small cubes using a single-use scalpel; one band was comprised of the gel at ∼18 kDa (where the major CaM band is located) and one was comprised of the gel between ∼10 and 17 kDa, the area under the major band. The rest of the in-gel digestion was performed as previously described in.^28^

##### Extraction of peptides and StageTip purification

StageTip extraction of peptides was performed as described previously in Rappsilber et al*.* (2007).^29^

##### nanoLC-MS/MS analyses

LC-MS analyses were performed as described previously with the following minor modifications.^30^ Peptide samples were separated on a EASY-nLC 1200 System (Thermo Fisher Scientific) using 20 cm long fused silica capillary columns (100 μm ID) packed with 3 μm 120 Å reversed phase C18 beads (Dr. Maisch, Ammerbuch, DE). The LC gradient was 90 min long with 5–35% B at 300 nL/min. LC solvent A was 0.1% (v/v) aq. acetic acid and LC solvent B was 20% 0.1% (v/v) acetic acid, 80% acetonitrile. MS data were collected with a Thermo Fisher Scientific Orbitrap Fusion Lumos Tribrid mass spectrometer. Data-dependent analysis was applied using Top15 selection with CID fragmentation.

##### Computation of MS raw files

Data.raw files were analyzed by MaxQuant/Andromeda^30^ version 1.5.2.8 using protein, peptide, and site FDRs of 0.01 and a score minimum of 40 for modified peptides, 0 for unmodified peptides; delta score minimum of 17 for modified peptides, 0 for unmodified peptides. MS/MS spectra were searched against the UniProt human database (updated July 22, 2015). MaxQuant search parameters: Variable modifications included Oxidation (M) and Phospho (S/T/Y). Carbamidomethyl (C) was a fixed modification. Maximum missed cleavages was 2, enzyme was Trypsin/P and max. mharge was 7. The MaxQuant “match between runs” feature was disabled. The initial search tolerance for FTMS scans was 20 ppm and 0.5 Da for ITMS MS/MS scans.

##### Semi-quantitative analysis of contaminants and CaM protein

The intensity of peptides annotated as CaM, keratin, or bacterial protein was summed up and their relative intensities were calculated by dividing them to the total peptide intensity in the samples.

#### Octet BioLayer interferometry (BLI) measurement

• Octet Red 96 (ForteBio, Pall Life Sciences)
• BSA, Sigma-Aldrich cat. no. A3294-100G
• Calcium chloride, Sigma-Aldrich cat. no. 746495-1 KG
• Tris-HCl, Sigma-Aldrich cat. no. T5941
• NaCl Sigma-Aldrich, # 746398-5 KG

The binding of His-CaM to FR or FL-MLCK-FLAG protein in the presence of Ca^2+^ was analyzed using the Octet Red 96 (ForteBio, Pall Life Sciences) following the manufacturer’s procedures in duplicates. The reaction was carried out in black 96 well plates maintained at 30 °C. The reaction volume was 200 μL in each well. The Octet buffer contained 20 mM Tris-HCl, 200 mM NaCl, and 0.1% BSA, pH 8.0. The Association buffer contained 20 mM Tris-HCl, 200 mM NaCl, 1.5 mM Ca^2+^ and 0.1% BSA, pH 8.0. The Dissociation buffer contained 20 mM Tris-HCl, 200 mM NaCl, and 0.1% BSA, pH 8.0. The concentration of ligand – His-CaM^WT^, His-CaM^21A,57A^ or His-CaM^94A,130A^ – in the Octet buffer was 2 μM. The concentration of His-GST as the quench in Octet Buffer was 0.68 μM. The concentration of FR or MLCK-FLAG as the analyte in the Association buffer was 1.5 μM. Ni-NTA optical probes were loaded with His-CaM^WT^, His-CaM^21A,57A^ or His-CaM^94A,130A^ as ligands for 110 s and quenched with His-GST for 80 s prior to binding analysis. While not loaded with ligand, the control probes were quenched with His-GST. In each experiment, “Control” refers to the analyte (either FR or MLCK-FLAG) binding to the ligand-free probe. This control is performed to demonstrate that the association seen in the ligand bound probes is not due to non-specific binding but is, in fact, specific. The binding of the analyte (either FR or MLCK-FLAG) to the optical probes was measured simultaneously using instrumental defaults for 236 s. The dissociation was measured for 287 s. There was no binding of FR to the unloaded probes; however, slight binding of MLCK-FLAG to the unloaded probes was observed. The data were analyzed by the Octet data analysis software. The association and dissociation curves for FR binding were globally fit with a 1:1 ligand model, and the curves for MLCK-FLAG binding were locally fit for 80s. The data were plotted using Prism 7.

#### DNA sequences

##### CaM sequences

**Bolding**: mutations.

##### His-CaM^WT^ DNA sequence (protein molecular weight: ∼19.8 kDa)

atgggcagcagccatcatcatcatcatcacagcagcggcctggtgccgcgcggcagccatagcgaaaacctctacttccaatcgatggctgaccagctgactgaggagcagattgcagagttcaaggaggccttctccctctttgacaaggatggagatggcactatcaccaccaaggagttggggacagtgatgagatccctgggacagaaccccactgaagcagagctgcaggatatgatcaatgaggtggatgcagatgggaacgggaccattgacttcccggagttcctgaccatgatggccagaaagatgaaggacacagacagtgaggaggagatccgagaggcgttccgtgtctttgacaaggatgggaatggctacatcagcgccgcagagctgcgtcacgtaatgacgaacctgggggagaagctgaccgatgaggaggtggatgagatgatcagggaggctgacatcgatggagatggccaggtcaattatgaagagtttgtacagatgatgactgcaaagtga

##### His-CaM^21A,57A^ DNA sequence (protein molecular weight: ∼19.8 kDa)

atgggcagcagccatcatcatcatcatcacagcagcggcctggtgccgcgcggcagccatagcgaaaacctctacttccaatcgatggctgaccagctgactgaggagcagattgcagagttcaaggaggccttctccctctttg**c**caaggatggagatggcactatcaccaccaaggagttggggacagtgatgagatccctgggacagaaccccactgaagcagagctgcaggatatgatcaatgaggtgg**ca**gcagatgggaacgggaccattgacttcccggagttcctgaccatgatggccagaaagatgaaggacacagacagtgaggaggagatccgagaggcgttccgtgtctttgacaaggatgggaatggctacatcagcgccgcagagctgcgtcacgtaatgacgaacctgggggagaagctgaccgatgaggaggtggatgagatgatcagggaggctgacatcgatggagatggccaggtcaattatgaagagtttgtacagatgatgactgcaaagtga

##### His-CaM^94A,130A^ DNA sequence (protein molecular weight: ∼19.8 kDa)

atgggcagcagccatcatcatcatcatcacagcagcggcctggtgccgcgcggcagccatagcgaaaacctctacttccaatcgatggctgaccagctgactgaggagcagattgcagagttcaaggaggccttctccctctttgacaaggatggagatggcactatcaccaccaaggagttggggacagtgatgagatccctgggacagaaccccactgaagcagagctgcaggatatgatcaatgaggtggatgcagatgggaacgggaccattgacttcccggagttcctgaccatgatggccagaaagatgaaggacacagacagtgaggaggagatccgagaggcgttccgtgtctttg**c**caaggatgggaatggctacatcagcgccgcagagctgcgtcacgtaatgacgaacctgggggagaagctgaccgatgaggaggtggatgagatgatcagggaggctg**c**catcgatggagatggccaggtcaattatgaagagtttgtacagatgatgactgcaaagtga.

##### His-CaM^21A,57A,94A,130A^ DNA sequence (protein molecular weight: ∼19.8 kDa)

atgggcagcagccatcatcatcatcatcacagcagcggcctggtgccgcgcggcagccatagcgaaaacctctacttccaatcgatggctgaccagctgactgaggagcagattgcagagttcaaggaggccttctccctctttg**c**caaggatggagatggcactatcaccaccaaggagttggggacagtgatgagatccctgggacagaaccccactgaagcagagctgcaggatatgatcaatgaggtgg**ca**gcagatgggaacgggaccattgacttcccggagttcctgaccatgatggccagaaagatgaaggacacagacagtgaggaggagatccgagaggcgttccgtgtctttg**c**caaggatgggaatggctacatcagcgccgcagagctgcgtcacgtaatgacgaacctgggggagaagctgaccgatgaggaggtggatgagatgatcagggaggctg**c**catcgatggagatggccaggtcaattatgaagagtttgtacagatgatgactgcaaagtga.

##### MLCK FRET REPORTER (FR) DNA sequence (protein molecular weight: ∼58.2 kDa)

• **Bolding**: EYFP
• ***Bold italics**: Calmodulin binding domain*
• Underlining: ECFP
• ***Bold underlined italics***: Flexible linker
• *Italics*: FLAG tag

**Atggtgagcaagggcgaggagctgttcaccggggtggtgcccatcctggtcgagctggacggcgacgtaaacggccacaagttcagcgtgtccggcgagggcgagggcgatgccacctacggcaagctgaccctgaagttcatctgcaccaccggcaagctgcccgtgccctggcccaccctcgtgaccaccttcggctacggcctgatgtgcttcgcccgctaccccgaccacatgcgccagcacgacttcttcaagtccgccatgcccgaaggctacgtccaggagcgcaccatcttcttcaaggacgacggcaactacaagacccgcgccgaggtgaagttcgagggcgacaccctggtgaaccgcatcgagctgaagggcatcgacttcaaggaggacggcaacatcctggggcacaagctggagtacaactacaacagccacaacgtctatatcatggccgacaagcagaagaacggcatcaaggtgaacttcaagatccgccacaacatcgaggacggcagcgtgcagctcgccgaccactaccagcagaacacccccatcggcaacggccccgtgctgctgcccgacaaccactacctgagctaccagtccgccctgagcaaagaccccaacgagaagcgcgatcacatggtcctgctggagttcgtgaccgccgccgggatcactctcggcatggacgagctgtacaag*ggtaccgccgctcgtcagaaatggcagaaaaccggacatgcggtgcgtgcgattggccgtctggctgctaccggt***agcaagggcgaggagctgttcaccggggtggtgcccatcctggtcgagctggacggcgacgtaaacggccacaggttcagcgtgtccggcgagggcgagggcgatgccacctacggcaagctgaccctgaagttcatctgcaccaccggcaagctgcccgtgccctggcccaccctcgtgaccaccctgacctggggcgtgcagtgcttcagccgctaccccgaccacatgaagcagcacgacttcttcaagtccgccatgcccgaaggctacgtccaggagcgtaccatcttcttcaaggacgacggcaactacaagacccgcgccgaggtgaagttcgagggcgacaccctggtgaaccgcatcgagctgaagggcatcgacttcaaggaggacggcaacatcctggggcacaagctggagtacaactacatcagccacaacgtctatatcaccgccgacaagcagaagaacggcatcaaggcccacttcaagatccgccacaacatcgaggacggcagcgtgcagctcgccgaccactaccagcagaacacccccatcggcgacggccccgtgctgctgcccgacaaccactacctgagcacccagtccgccctgagcaaagaccccaacgagaagcgcgatcacatggtcctgctggagttcgtgaccgccgccgggatcactctcggcatggacgagctgtacaag***cccgggggtggatctggtggatctggtggatctatg****gattacaaggatgacgatgacaag*.

##### Full-length MLCK DNA sequence (protein molecular weight: ∼212.0 kDa)

• ***Bold italics***: Calmodulin binding domain
• ***Bold underlined italics***: Flexible linker
• *Italics*: FLAG tag

atgggggatgtgaagctggttgcctcgtcacacatttccaaaacctccctcagtgtggatccctcaagagttgactccatgcccctgacagaggcccctgctttcattttgccccctcggaacctctgcatcaaagaaggagccaccgccaagttcgaagggcgggtccggggttacccagagccccaggtgacatggcacagaaacgggcaacccatcaccagcgggggccgcttcctgctggattgcggcatccgggggactttcagccttgtgattcatgctgtccatgaggaggacaggggaaagtatacctgtgaagccaccaatggcagtggtgctcgccaggtgacagtggagttgacagtagaaggaagttttgcgaagcagcttggtcagcctgttgtttccaaaaccttaggggatagattttcagcttcagcagtggagacccgtcctagcatctggggggagtgcccaccaaagtttgctaccaagctgggccgagttgtggtcaaagaaggacagatgggacgattctcctgcaagatcactggccggccccaaccgcaggtcacctggctcaagggaaatgttccactgcagccgagtgcccgtgtgtctgtgtctgagaagaacggcatgcaggttctggaaatccatggagtcaaccaagatgacgtgggagtgtacacgtgcctggtggtgaacgggtcggggaaggcctcgatgtcagctgaactttccatccaaggtttggacagtgccaataggtcatttgtgagagaaacaaaagccaccaattcagatgtcaggaaagaggtgaccaatgtaatctcaaaggagtcgaagctggacagtctggaggctgcagccaaaagcaagaactgctccagcccccagagaggtggctccccaccctgggctgcaaacagccagcctcagcccccaagggagtccaagctggagtcatgcaaggactcgcccagaacggccccgcagactccggtccttcagaagacttccagctccatcaccctgcaggccgcaagagttcagccggaaccaagagcaccaggcctgggggtcctatcaccttctggagaagagaggaagaggccagctcctccccgtccagccaccttccccaccaggcagcctggcctggggagccaagatgttgtgagcaaggctgctaacaggagaatccccatggagggccagagggattcagcattccccaaatttgagagcaagccccaaagccaggaggtcaaggaaaatcaaactgtcaagttcagatgtgaagtttccgggattccaaagcctgaagtggcctggttcctggaaggcacccccgtgaggagacaggaaggcagcattgaggtttatgaagatgctggctcccattacctctgcctgctgaaagcccggaccagggacagtgggacatacagctgcactgcttccaacgcccaaggccaggtgtcctgtagctggaccctccaagtggaaaggcttgccgtgatggaggtggccccctccttctccagtgtcctgaaggactgcgccgttattgagggccaggattttgtgctgcagtgctccgtacgggggaccccagtgccccggatcacttggctgctgaatgggcagcccatccagtacgctcgctccacctgcgaggccggcgtggctgagctccacatccaggatgccctgccggaggaccatggcacctacacctgcctagctgagaatgccttggggcaggtgtcctgcagcgcctgggtcaccgtccatgaaaagaagagtagcaggaagagtgagtaccttctgcctgtggctcccagcaagcccactgcacccatcttcctgcagggcctctctgatctcaaagtcatggatggaagccaggtcactatgactgtccaagtgtcagggaatccaccccctgaagtcatctggctgcacaatgggaatgagatccaagagtcagaggacttccactttgaacagagaggaactcagcacagcctttgtatccaggaagtgttcccggaggacacgggcacgtacacctgcgaggcctggaacagcgctggagaggtccgcacccaggccgtgctcacggtacaagagcctcacgatggcacccagccctggttcatcagtaagcctcgctcagtgacagcctccctgggccagagtgtcctcatctcctgcgccatagctggtgacccctttcctaccgtgcactggctcagagatggcaaagccctctgcaaagacactggccacttcgaggtgcttcagaatgaggacgtgttcaccctggttctaaagaaggtgcagccctggcatgccggccagtatgagatcctgctcaagaaccgggttggcgaatgcagttgccaggtgtcactgatgctacagaacagctctgccagagcccttccacgggggagggagcctgccagctgcgaggacctctgtggtggaggagttggtgctgatggtggtggtagtgaccgctatgggtccctgaggcctggctggccagcaagagggcagggttggctagaggaggaagacggcgaggacgtgcgaggggtgctgaagaggcgcgtggagacgaggcagcacactgaggaggcgatccgccagcaggaggtggagcagctggacttccgagacctcctggggaagaaggtgagtacaaagaccctatcggaagacgacctgaaggagatcccagccgagcagatggatttccgtgccaacctgcagcggcaagtgaagccaaagactgtgtctgaggaagagaggaaggtgcacagcccccagcaggtcgattttcgctctgtcctggccaagaaggggacttccaagacccccgtgcctgagaaggtgccaccgccaaaacctgccaccccggattttcgctcagtgctgggtggcaagaagaaattaccagcagagaatggcagcagcagtgccgagaccctgaatgccaaggcagtggagagttccaagcccctgagcaatgcacagccttcagggcccttgaaacccgtgggcaacgccaagcctgctgagaccctgaagccaatgggcaacgccaagcctgccgagaccctgaagcccatgggcaatgccaagcctgatgagaacctgaaatccgctagcaaagaagaactcaagaaagacgttaagaatgatgtgaactgcaagagaggccatgcagggaccacagataatgaaaagagatcagagagccaggggacagccccagccttcaagcagaagctgcaagatgttcatgtggcagagggcaagaagctgctgctccagtgccaggtgtcttctgaccccccagccaccatcatctggacgctgaacggaaagaccctcaagaccaccaagttcatcatcctctcccaggaaggctcactctgctccgtctccatcgagaaggcactgcctgaggacagaggcttatacaagtgtgtagccaagaatgacgctggccaggcggagtgctcctgccaagtcaccgtggatgatgctccagccagtgagaacaccaaggccccagagatgaaatcccggaggcccaagagctctcttcctcccgtgctaggaactgagagtgatgcgactgtgaaaaagaaacctgcccccaagacacctccgaaggcagcaatgccccctcagatcatccagttccctgaggaccagaaggtacgcgcaggagagtcagtggagctgtttggcaaagtgacaggcactcagcccatcacctgtacctggatgaagttccgaaagcagatccaggaaagcgagcacatgaaggtggagaacagcgagaatggcagcaagctcaccatcctggccgcgcgccaggagcactgcggctgctacacactgctggtggagaacaagctgggcagcaggcaggcccaggtcaacctcactgtcgtggataagccagaccccccagctggcacaccttgtgcctctgacattcggagctcctcactgaccctgtcctggtatggctcctcatatgatgggggcagtgctgtacagtcctacagcatcgagatctgggactcagccaacaagacgtggaaggaactagccacatgccgcagcacctctttcaacgtccaggacctgctgcctgaccacgaatataagttccgtgtacgtgcaatcaacgtgtatggaaccagtgagccaagccaggagtctgaactcacaacggtaggagagaaacctgaagagccgaaggatgaagtggaggtgtcagatgatgatgagaaggagcccgaggttgattaccggacagtgacaatcaatactgaacaaaaagtatctgacttctacgacattgaggagagattaggatctgggaaatttggacaggtctttcgacttgtagaaaagaaaactcgaaaagtctgggcagggaagttcttcaaggcatattcagcaaaagagaaagagaatatccggcaggagattagcatcatgaactgcctccaccaccctaagctggtccagtgtgtggatgcctttgaagaaaaggccaacatcgtcatggtcctggagatcgtgtcaggaggggagctgtttgagcgcatcattgacgaggactttgagctgacggagcgtgagtgcatcaagtacatgcggcagatctcggagggagtggagtacatccacaagcagggcatcgtgcacctggacctcaagccggagaacatcatgtgtgtcaacaagacgggcaccaggatcaagctcatcgactttggtctggccaggaggctggagaatgcggggtctctgaaggtcctctttggcaccccagaatttgtggctcctgaagtgatcaactatgagcccatcggctacgccacagacatgtggagcatcggggtcatctgctacatcctagtcagtggcctttcccccttcatgggagacaacgataacgaaaccttggccaacgttacctcagccacctgggacttcgacgacgaggcattcgatgagatctccgacgatgccaaggatttcatcagcaatctgctgaagaaagatatgaaaaaccgcctggactgcacgcagtgccttcagcatccatggctaatgaaagataccaagaacatggaggccaagaaactctccaaggaccggatgaagaagtacatggca***agaaggaaatggcagaaaacgggcaatgctgtgagagccattggaagactgtcctctatg***gcaatgatctcagggctcagtggcaggaaatcctcaacagggtcaccaaccagcccgctcaatgcagaaaaactagaatctgaagaagatgtgtcccaagctttccttgaggctgttgctgaggaaaagcctcatgtaaaaccctatttctctaagaccattcgcgatttagaagttgtggagggaagtgctgctagatttgactgcaagattgaaggatacccagaccccgaggttgtctggttcaaagatgaccagtcaatcagggagtcccgccacttccagatagactacgatgaggacgggaactgctctttaattattagtgatgtttgcggggatgacgatgccaagtacacctgcaaggctgtcaacagtcttggagaagccacctgcacagcagagctcattgtggaaacgatggaggaaggtgaaggggaaggggaagaggaagaagag***gtcgacatg****gactacaaggacgacgacgacaag*.

### Quantification and statistical analysis

#### Analysis of CaM-FR binding assay (Figure 3)

For CaM^WT^, each data point is presented as the mean ± SD of 15 independent experiments. For CaM^21A,57A^, each data point is presented as mean ± SD of 16 independent experiments. For CaM^94A,130A^, each data point is presented as mean ± SD of 8 independent experiments, and for CaM^21A,57A,94A,130A^ each data point is presented as mean ± SD of 3 independent experiments. For reporter-only data, each data point is presented as a mean ± SD of 13 independent experiments. Curve fits were computed using the MATLAB (version R2020a) package doseResponse. Algebraic calculations involving the mathematical models were done in Wolfram Mathematica (version 12.1).

#### Comparison of on-bead and FRET-based binding assessment between FR and CaM^WT^ as a function of [Ca^2+^] (Figure S6)

Fraction of calmodulin bound to FR was quantified using ImageJ; GraphPad Prism 7 was used to plot mean and SD.

#### Bio-Layer interferometry (BLI) binding assays (Figure 5)

Following data collection, the data were analyzed using the Octet data analysis software (Octet Analysis Studio) and plotted using GraphPad Prism 7.

#### CaM-FR binding sensitivity assay (Figure S7)

Following the determination of the mean and SD using GraphPad Prism, a one-tailed Mann-Whitney *U* test was performed to determine the significance (*p* value) of the ratio increase.

#### High concentration CaM^WT^-FR binding quantification (Figure S8)

Following the determination of the mean and SD using GraphPad Prism, a one-tailed Mann-Whitney *U* test was performed to determine the significance (*p* value) of the ratio increase.

#### Impact of buffer addition on free [Ca^2+^] (Figure S10)

Following the determination of the mean and SD using GraphPad Prism, a two-tailed Mann-Whitney *U* test was performed to determine the significance (*p* value) of the fluorescence change.

#### Statistical analysis

Statistical calculations were performed using MATLAB. AUC was computed using the trapz built-in MATLAB function.

## Data Availability

• The original data reported in this paper will be shared by the lead contact upon request. • Code for reproducing the mathematical modeling and statistical analysis of binding is freely and publicly available at Open Science Foundation: https://osf.io/zt4rk/. • Any additional information required to reanalyze the data reported in this paper is available from the lead contact upon request.
