# Supplementary material for: Mathematical modeling and biochemical analysis support partially ordered calmodulin-myosin light chain kinase binding
Source: iScience. 2023 Feb 4;26(4):106146. doi: 10.1016/j.isci.2023.106146 (PMC10031086; doi:10.1016/j.isci.2023.106146)
Supplement: Document S1. Figures S1–S10 [file mmc1.pdf]

iScience, Volume *26*

Supplemental information

Mathematical modeling and biochemical analysis support partially ordered calmodulin-myosin light chain kinase binding

Melissa J.S. MacEwen, Domnita-Valeria Rusnac, Henok Ermias, Timothy M. Locke, Hayden E. Gizinski, Joseph P. Dexter, and Yasemin Sancak

iScience, Volume ■ ■

Supplemental information

Mathematical modeling and biochemical analysis support partially ordered calmodulin-myosin light chain kinase binding

Melissa J.S. MacEwen, Domnita-Valeria Rusnac, Henok Ermias, Timothy M. Locke, Hayden E. Gizinski, Joseph P. Dexter, and Yasemin Sancak

SUPPLEMENTAL FIGURE 1

A

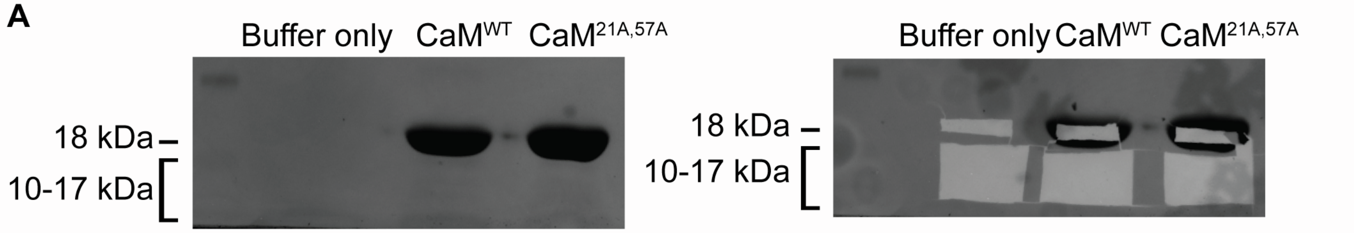

B

	% CaM intensity			% Keratin, E.coli proteins		
Molecular Weight	Buffer only	CaM^{WT}	CaM^{21A,57A}	Buffer only	CaM^{WT}	CaM^{21A,57A}
18 kDa	17%	96%	99%	34%	0%	0%
10-17 kDa	53%	91%	66%	11%	3%	15%
Lane total	51%	96%	96%	13%	1%	2%

Supplemental Figure 1: Mass spectrometry analysis of purified calmodulin proteins, Related to Figure 2. Semi-quantitative mass spectrometry was performed to assess the purity of purified CaM^{WT} and CaM^{21A,57A} proteins; a "loading buffer only" lane was used as a control. **(A)** Following SDS-PAGE, the gel was Coomassie stained, and bands of interest were excised and analyzed by mass spectrometry. (**B**) The identified peptides were categorized as CaM or a contaminating protein (keratin or *E. coli* protein) using their annotations. Finally, the percent abundance of either CaM or contaminants in each band was determined using their intensity in mass spectrometry data.

SUPPLEMENTAL FIGURE 2

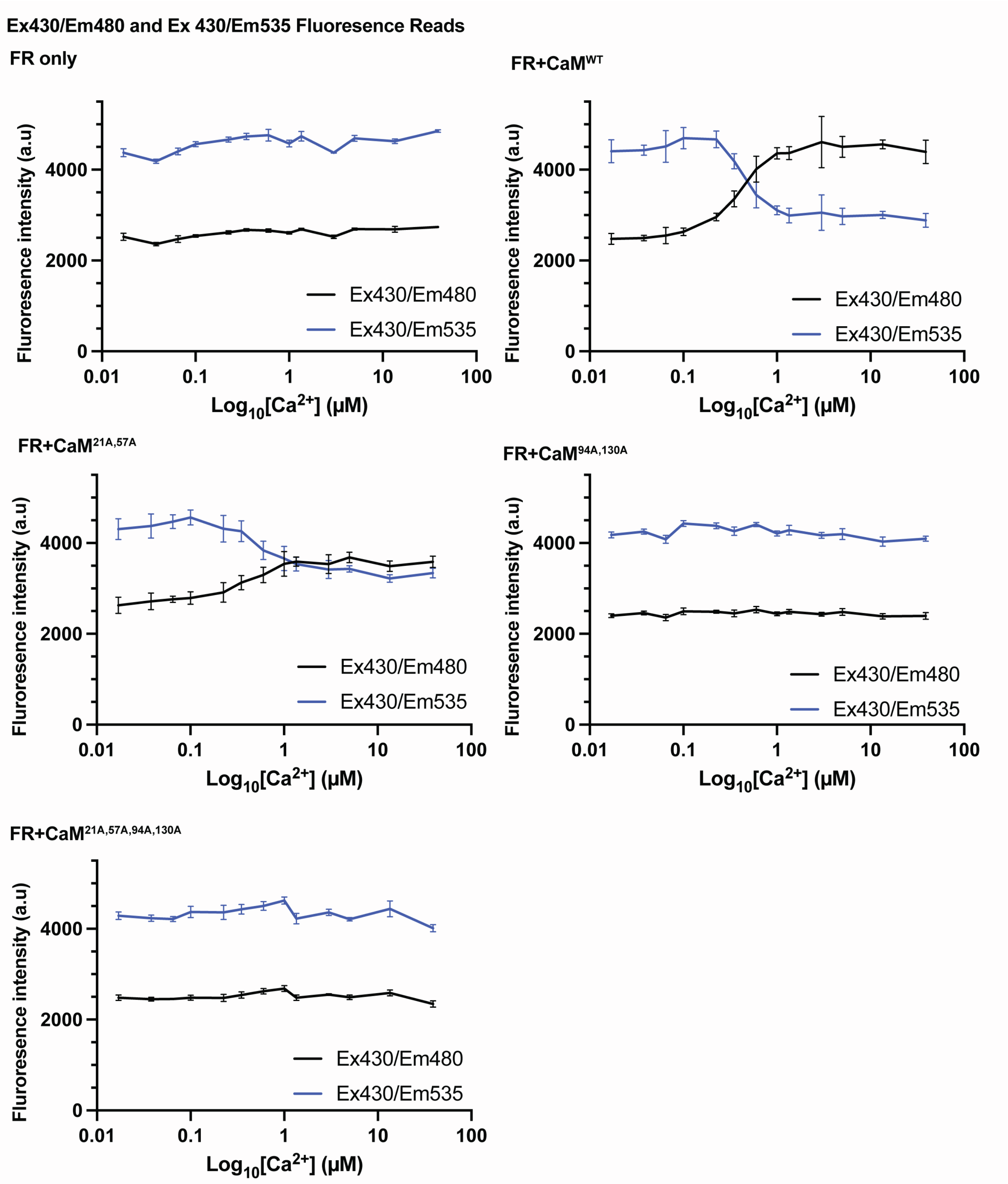

Supplemental Figure 2: Fluorescence intensities of CaM-FR binding assay, Related to Figure 3. For each of the indicated CaM-FR combinations, 673 nM CaM and 22.9 nM FR were combined in 150 µl of Ca^{2+} buffer (14 conditions between 0 µM and 39 µM). Fluorescence intensity of [Ex 430, Em 480], followed by intensity of [Ex 430, Em 535], was measured using a plate reader. Shown are mean ± standard deviation of raw data from at least 5 replicates for CaM^{WT}, $CaM^{21A,57A}$, and $CaM^{94A,130A}$, and 3 replicates for $CaM^{21A,57A,94A,130A}$ and FR only. **Fig. 3** shows the full dataset following analysis.

SUPPLEMENTAL FIGURE 3

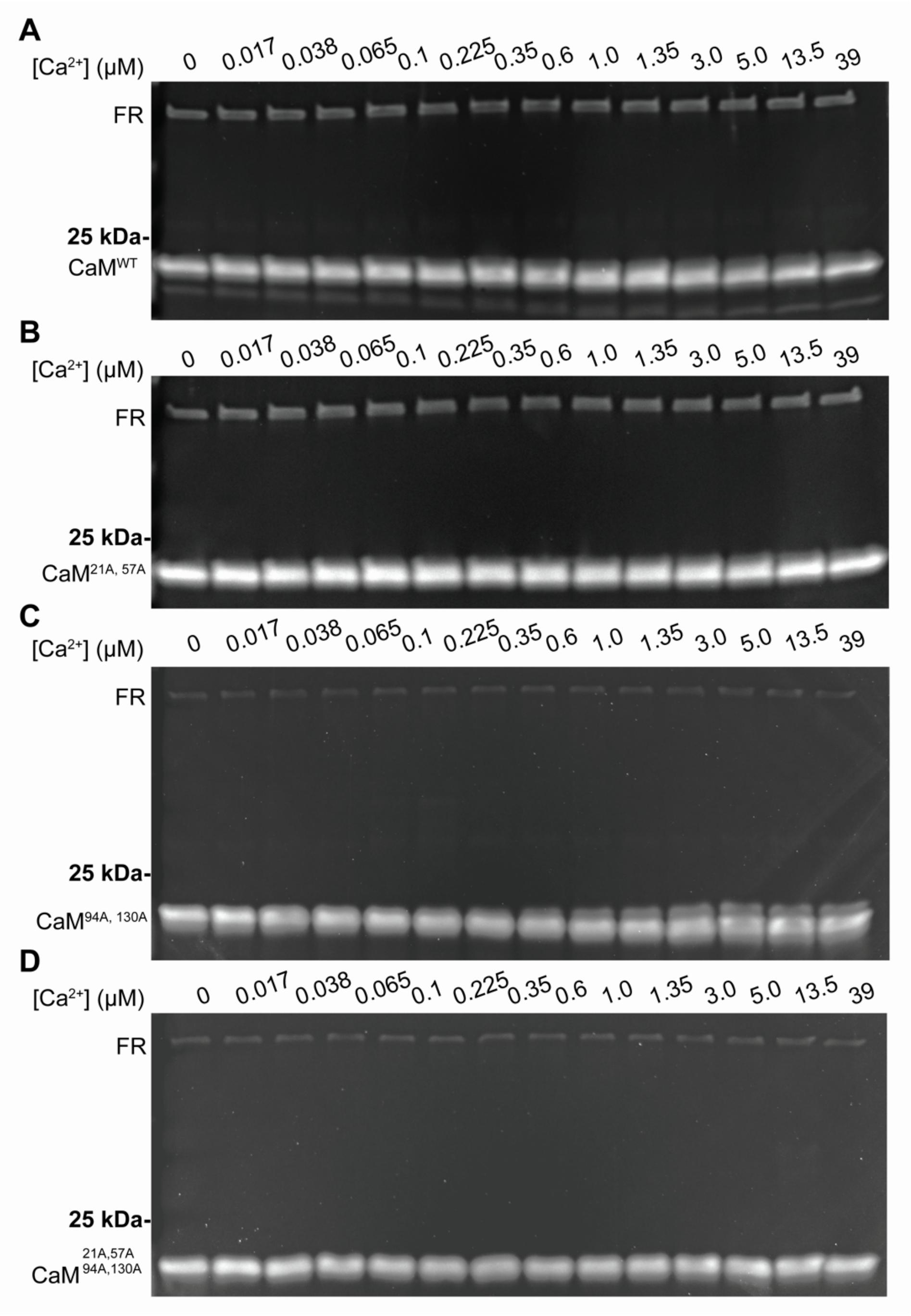

Supplemental Figure 3: Purified FR and CaM used in data collection, Related to Figure 3. A portion of each sample for fluorescent detection of FR-CaM binding was recovered from the wells. SDS-PAGE and SYPRO™ Ruby staining confirmed the presence of excess CaM compared to FR in the FRET-based binding assay for **(A)** CaM^{WT} and FR, **(B)** $CaM^{21A,57A}$ and FR, **(C)** $CaM^{94A,130A}$ and FR, and **(D)** $CaM^{21A,57A,94A,130A}$.

SUPPLEMENTAL FIGURE 4

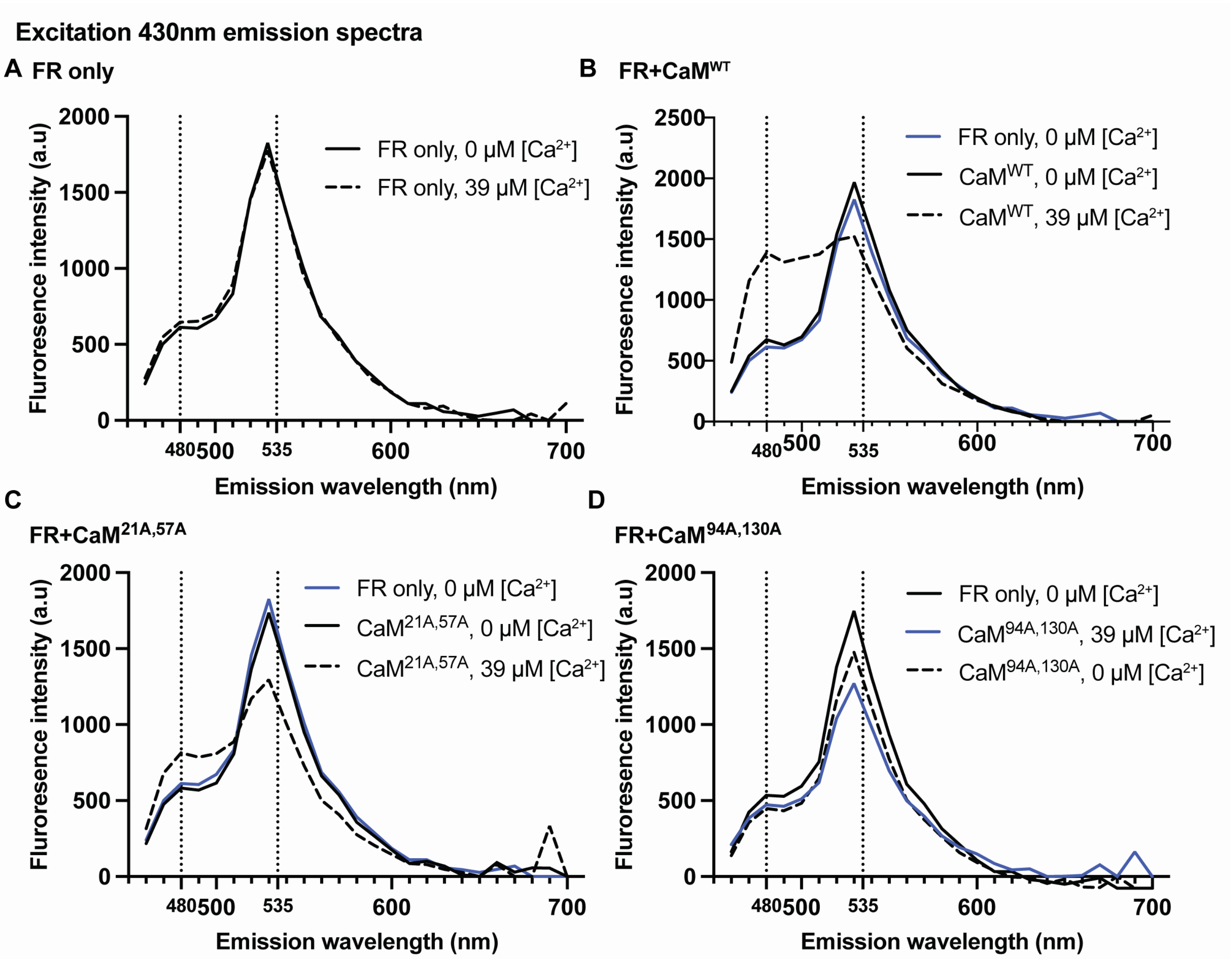

Supplemental Figure 4. Emission spectra of FR at Ex 430, Related to Figure 3. The emission spectra (460 nm - 700 nm, 10 nm steps) of **(A)** FR alone or with **(B)** CaM^{WT}, **(C)** $CaM^{21A,57A}$, or **(D)** $CaM^{94A,130A}$ was at either 0 or 39 μM [Ca^{2+}]. Dotted lines indicate the Em 480 and Em 535 wavelengths, which were used in FRET-based FR-CaM binding assays. One representative read for each condition is shown. FR-only spectra are included in the CaM graphs as a reference.

SUPPLEMENTAL FIGURE 5

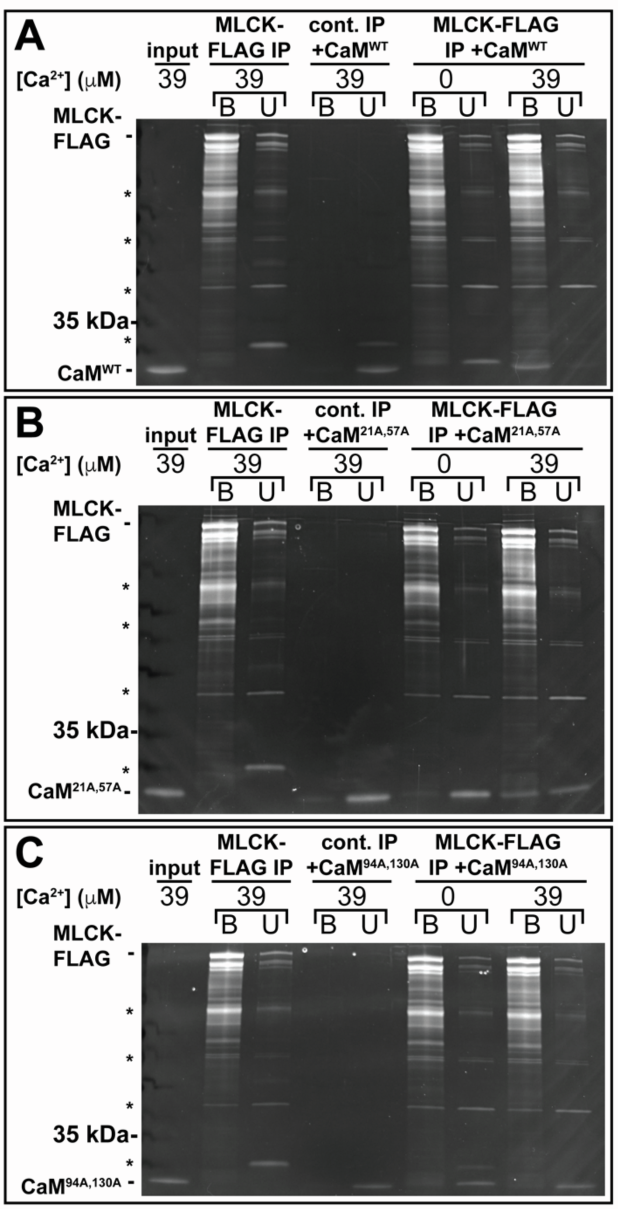

Supplemental Figure 5: Purified CaM^{WT} and CaM^{21A,57A} show Ca^{2+}-dependent interaction with MLCK-FLAG; CaM^{94A,130A} does not, Related to Figure 4. Representative images showing binding between bead-immobilized MLCK-FLAG and **(A)** CaM^{WT}, **(B)** CaM^{21A,57A}, and **(C)** CaM^{94A,130A} in buffers with the indicated free [Ca^{2+}]. Unbound and bound fractions were analyzed by SDS-PAGE followed by SYPRO^{TM} Ruby protein gel stain. MLCK-FLAG without CaM addition and a control binding experiment using non-transfected HEK 293T cells serve as controls. The input lane shows the purified CaM^{WT}, CaM^{21A,57A}, or CaM^{94A,130A} added to the on-bead binding assay. Asterisks (*) indicates background bands.

SUPPLEMENTAL FIGURE 6

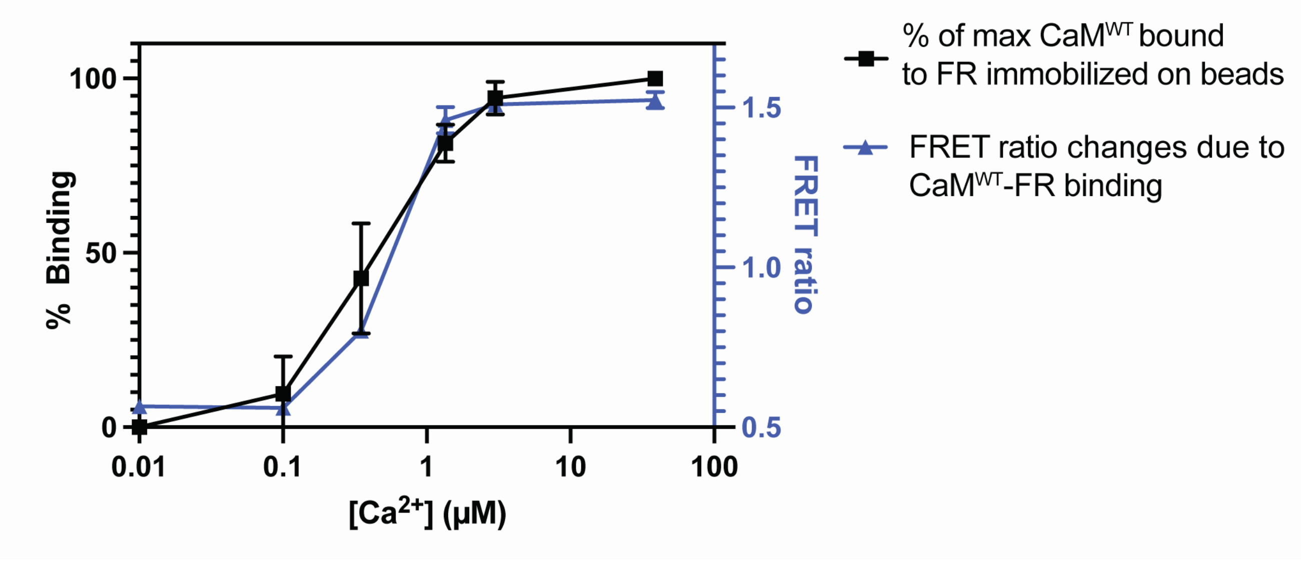

Supplemental Figure 6. Comparison of on-bead and FRET-based binding assessment between FR and CaM^{WT} as a function of [Ca^{2+}], Related to Figure 4. Data collected using FRET-based binding assay for CaM^{WT}-FR was plotted alongside data collected using on-bead binding assay. To quantify the relative amount of CaM bound to FR, CaM^{WT}-FR binding at 39 μM [Ca^{2+}] was set to 100%, binding at other [Ca^{2+}] relative to this maximum signal were calculated. Two replicates of images of on-bead binding were quantified using ImageJ; mean and ± standard deviation are shown. 15 replicates of FRET ratio changes due to CaM^{WT}-FR binding are shown as mean ± standard deviation.

SUPPLEMENTAL FIGURE 7

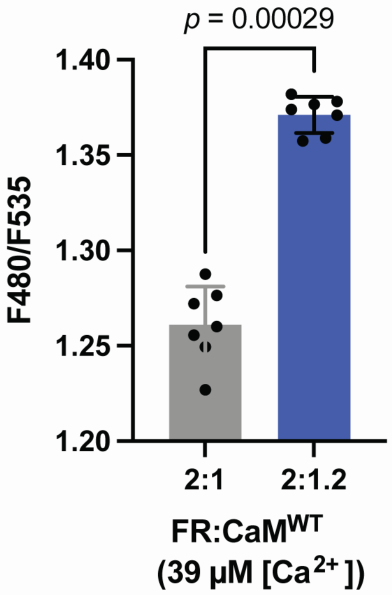

Supplemental Figure 7: CaM-FR binding assay is sensitive enough to detect a calculated 8.5% increase in CaM-FR binding, Related to Figure 5. A modified version of the CaM-FR FRET-based binding assay using a FR:CaM^{WT} ratio of either 2:1 or 2:1.2 (22.9 nM FR and 11.45 nM or 13.75 nM CaM) in high [Ca^{2+}] was performed. The Em 480/Em F535 ratio increased significantly between conditions (p = 0.00029 by a one-tailed Mann-Whitney *U* test), from an average of 1.26 to 1.37 (an 8.7% increase in FRET ratio). Assuming Model 2 and the reference parameter values from Fajmut, Brumen *et al*. [9], the fraction of FR bound to CaM^{WT} is predicted to differ by 8.5% between the two conditions (46.2% vs. 54.7% bound). Mean ± standard deviation of 7 replicates for each condition are shown.

SUPPLEMENTAL FIGURE 8

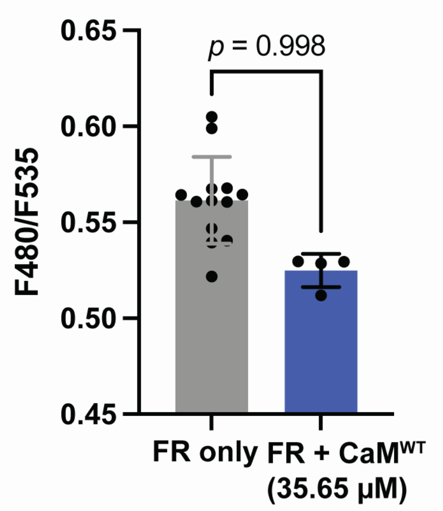

Supplemental Figure 8: CaM^{WT} does not bind to FR in the absence of Ca^{2+} even at high concentrations, Related to Figure 5. To test the robustness of our observation of no binding in zero [Ca^{2+}], we repeated the FRET-based binding assay with 35.65 μM CaM^{WT}, for which Model 1 predicts 21.7% binding even if previous estimates of a key parameter value are incorrect by an order of magnitude. CaM^{WT} was concentrated to 66.6 μg/μl so that the volume of protein solution used was comparable to previous assays. As in the original assay, the F480/F535 ratio was not higher than baseline (p = 0.998 by a one-tailed Mann-Whitney U test). Mean ± standard deviation of 13 replicates for FR only read and 4 replicates for FR+ CaM^{WT} reads are shown.

SUPPLEMENTAL FIGURE 9

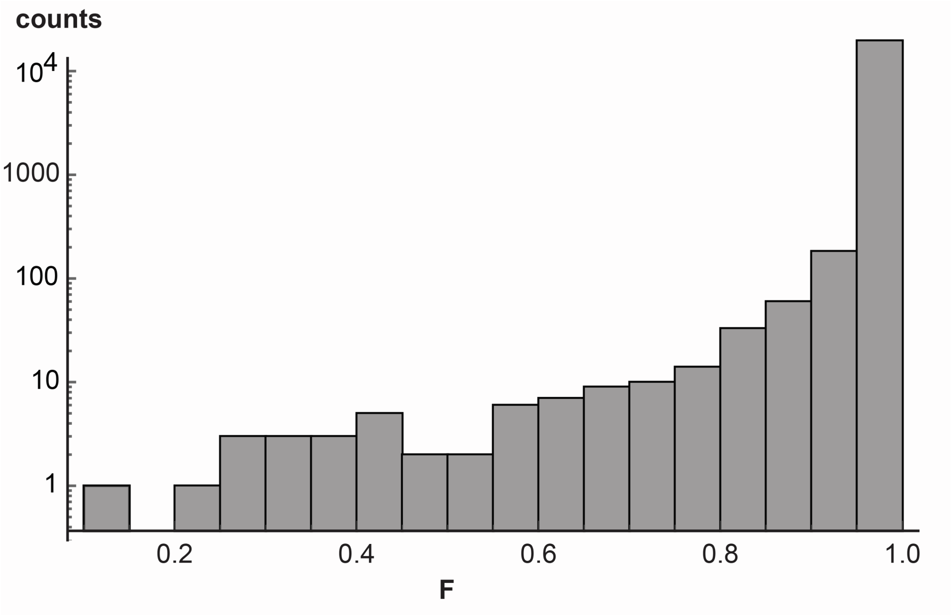

Supplemental Figure 9. Histogram from sensitivity analysis of model of $CaM^{21A,57A}$, Related to Figure 5. The histogram shows the distribution of predicted binding fractions for 200,000 combinations of K_4 and K_6 chosen at random from the interval [0.01v, 100v], where v is the reference value from Fajmut, Brumen *et al*.[9]

SUPPLEMENTAL FIGURE10

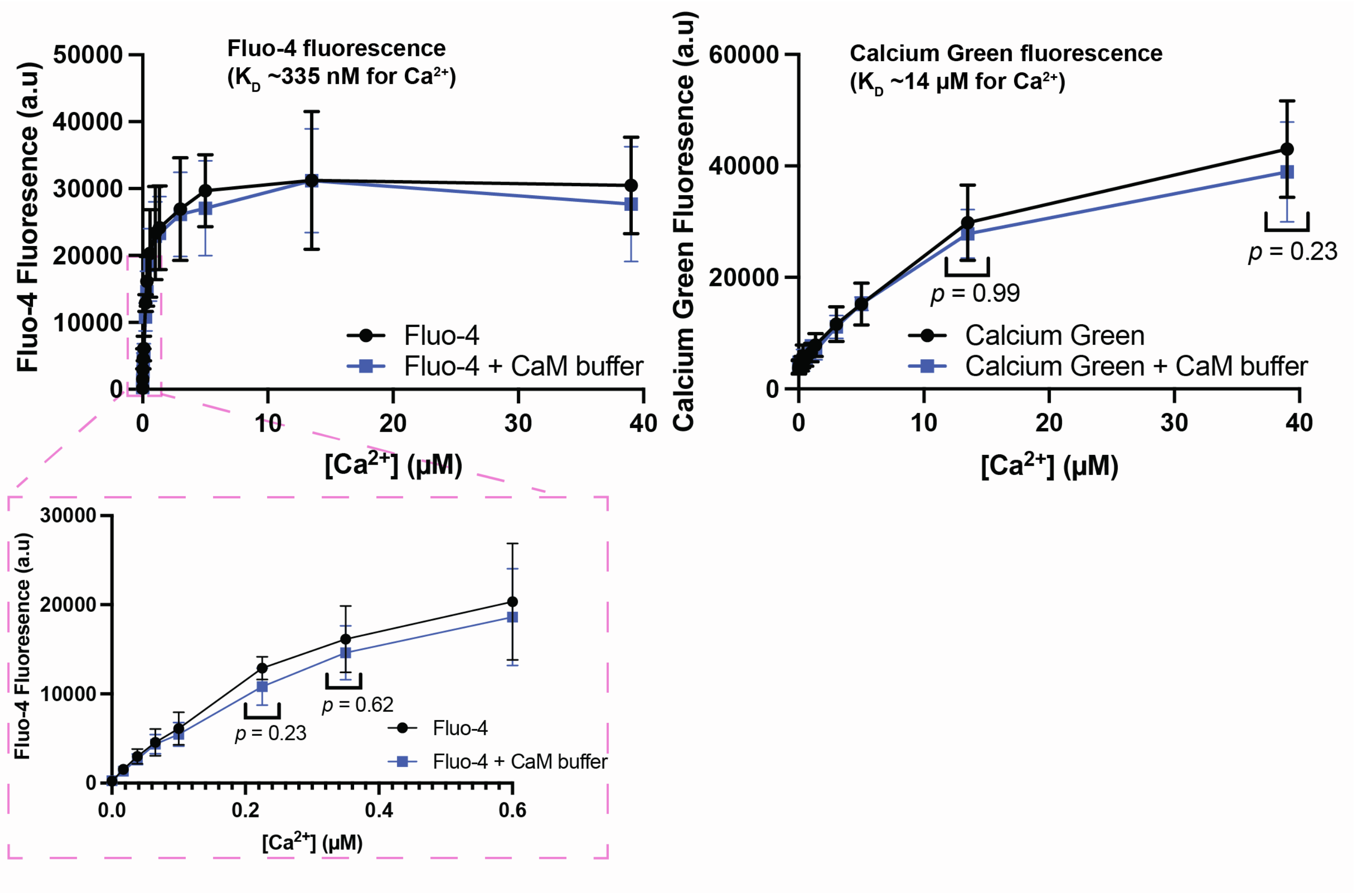

Supplemental Figure 10. Buffer addition to FR-CaM binding assay does not change assay free [Ca^{2+}], Related to Figure 5. Ca^{2+} indicator dyes Fluo-4 or Calcium Green were added to the 14-buffer series of the FR-CaM binding assay; data was collected using a plate reader with a GFP filter. The same buffer/Ca^{2+} indicator dye combinations were then assayed following the addition of a "dummy buffer" composed of the buffer in which all CaM proteins were stored. The area of interest (magnified region in red dotted line) highlights the data corresponding to [Ca^{2+}] most relevant to Fluo-4, which has K_D~335 nM. Calcium Green has K_D~14 μM. Mean ± standard deviation of four replicates for each condition are shown. *P* values were calculated using a two-tailed Mann-Whitney *U* test.
